# Early Triassic wrinkle structures on land: stressed environments and oases for life

**DOI:** 10.1038/srep10109

**Published:** 2015-06-09

**Authors:** Daoliang Chu, Jinnan Tong, Haijun Song, Michael J. Benton, David J. Bottjer, Huyue Song, Li Tian

**Affiliations:** 1 State Key Laboratory of Biogeology and Environmental Geology, China University of Geosciences, Wuhan 430074, China; 2 School of Earth Sciences, University of Bristol, Bristol, BS8 1RJ, UK; 3 Department of Earth Sciences, University of Southern California, Los Angeles, CA 90089, USA

## Abstract

Wrinkle structures in rocks younger than the Permian-Triassic (P-Tr) extinction have been reported repeatedly in marine strata, but rarely mentioned in rocks recording land. Here, three newly studied terrestrial P-Tr boundary rock succession in North China have yielded diverse wrinkle structures. All of these wrinkles are preserved in barely bioturbated shore-shallow lacustrine siliciclastic deposits of the Liujiagou Formation. Conversely, both the lacustrine siliciclastic deposits of the underlying Sunjiagou Formation and the overlying Heshanggou Formation show rich bioturbation, but no wrinkle structures or other microbial-related structures. The occurrence of terrestrial wrinkle structures in the studied sections reflects abnormal hydrochemical and physical environments, presumably associated with the extinction of terrestrial organisms. Only very rare trace fossils occurred in the aftermath of the P-Tr extinction, but most of them were preserved together with the microbial mats. This suggests that microbial mats acted as potential oases for the surviving aquatic animals, as a source of food and oxygen. The new finds suggests that extreme environmental stresses were prevalent both in the sea and on land through most of the Early Triassic.

In ancient siliciclastic and carbonate depositional systems, the interaction between microbial activities and physical sedimentary processes can produce microbially related sedimentary structures. These were widespread during the Proterozoic, and even extend back to the Early Archean^1^,^2^,^3^,^4^, but became restricted to highly stressed environments after the Cambrian^4^,^5^ because they were generally destroyed by burrowing organisms. In addition, occurrences of microbially related sedimentary structures provide the most valuable evidence for detecting early life and reestablishment of environments in the Precambrian^6^.

After the Cambrian, the most widespread microbially related sedimentary structures occur in shallow marine settings following the largest mass extinction of all time near the P-Tr boundary^7^,^8^,^9^. The P-Tr mass extinction event wiped out over 90% of marine species, and most terrestrial vertebrate and plant species^10^. The diversity of marine invertebrates declined dramatically^11^, and bioturbation returned to Precambrian levels^12^. Microbial buildups and mats dominated the seabed after the P-Tr crisis, in the form of microbialites^7^,^9^ and wrinkle structures^8^,^9^. Cyanobacteria- dominated marine ecosystems lasted for about 5 Myr in the Early Triassic, linked to the depression of bioturbation and/or to long-term unusual environmental conditions^9^.

Current work suggests that the destruction of terrestrial ecosystems happened simultaneously with the decline in marine diversity during the P-Tr transition^13^,^14^. However, less is known about the specific changeover of terrestrial ecosystems during the P-Tr transition. So far, most attention has focused on tetrapods and plants^15^,^16^. Recent reviews indicate that tetrapods on land suffered a massive loss during the P-Tr crisis, as severe as that of life in the oceans, but the extinction seems to have been less profound for plants and insects^17^. Here we present new observations and investigations of wrinkle structures and trace fossils as well as their space relations in shallow-shore lacustrine siliciclastic deposits of Lower Triassic strata from North China that provides an exceptionally detailed record of terrestrial aquatic ecosystems during the P-Tr crisis.

## Results

**Figure 1:**
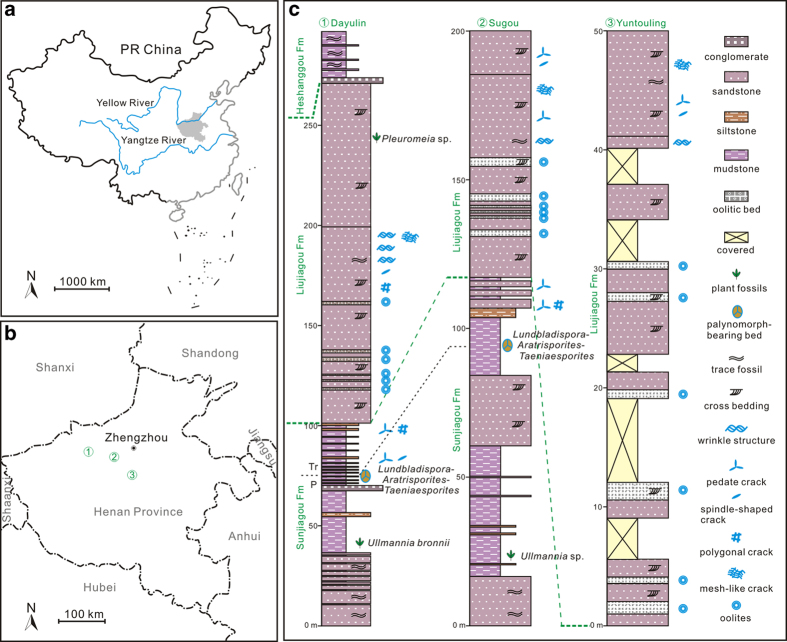
Maps and columnar sections. (**a**) Sketch map of China, showing Henan Province straddling the Yellow River (grey shading). (**b**) Locations of the studied sections, in Henan Province. (**c**) Stratigraphic sections showing lithologies and distributions of wrinkle structures; symbols (right) indicate major lithologies and fossils, and sedimentary structures (the nine symbols indicated in light blue). Abbreviations: Fm, Formation; P, Permian; Tr, Triassic. D.L.C. created the figure using MapGIS6.7 and CorelDRAW14.

North China was located in the north tropical zone, between about 10°N and 20°N during the P-Tr transitional time (see Supplementary Fig. S1). Since the Middle Permian, North China had been largely land, and rock successions are characterized by fluvial-lacustrine sedimentary facies. During the earliest Triassic, the central part of North China was dominated by east-west distributed lacustrine deposition, surrounded by dozens of lake deltas and flooding sandy plains (Supplementary Fig. S2b). The Upper Permian to Lower Triassic sedimentary sequence in North China is represented by the Shiqianfeng Group, which comprises, from oldest to youngest, the Sunjiagou, Liujiagou, and Heshanggou formations. Wrinkle structures are observed in the Liujiagou Formation in the Dayulin, Sugou, and Yuntouling sections, located within 100 km of each other in the central part of North China (Fig. 1).

The lower to middle part of the Sunjiagou Formation is composed of fine-grained sandstones and thinly interbedded siltstones, which show rich and intensive bioturbation, all deposited in a shallow-shore lake environment. The upper part of the Sunjiagou Formation is composed of mudstones, siltstones and fine-grained sandstones. Typical Late Permian plant fossil elements assigned to *Ullmannia* occur at the upper part of the Sunjiagou Formation in the Dayulin and Sugou sections. The P-Tr boundary is identified at a horizon about 20 m below the top of the Sunjiagou Formation by the occurrence of the *Lundbladispora*-*Aratrisporites*-*Taeniaesporites* assemblage^18^, all of these representing typical earliest Triassic palynomorphs^19^. The Liujiagou Formation (100–150 m in thickness) consists of fine-grained sandstones with abundant ripple marks and cross beddings, bearing diverse wrinkle structures but infrequent bioturbation, deposited in a lake shore environment. Plant fossils assigned to *Pleuromeia*, a typical Early Triassic element^20^, occur in the upper part of the Liujiagou Formation in the Dayulin section, and are sporadically distributed more widely within the Lower Triassic strata in North China^21^. In addition, oolitic beds accompanied with hummocky cross bedding, flat-pebble conglomerates and desiccation cracks with distinct geometries are observed in the Liujiagou Formation. The Heshanggou Formation (200–250 m in thickness) is mainly composed of purple siltstones and mudstones with diverse trace fossils, such as *Planolites*, *Psilonichnus*, *Scoyenia*, *Skolithos*, and *Taenidium*, indicating a shallow-shore lake palaeoenvironment. The bioturbation is well developed in the Heshanggou Formation, with a maximum measured bioturbation index^22^ up to 5, but no microbial-related structures have been observed. The overlying Middle Triassic Ermaying Formation (450–600 m in thickness) consists of sandstones and mudstones with abundant invertebrate fossils and diverse trace fossils.

**Figure 2:**
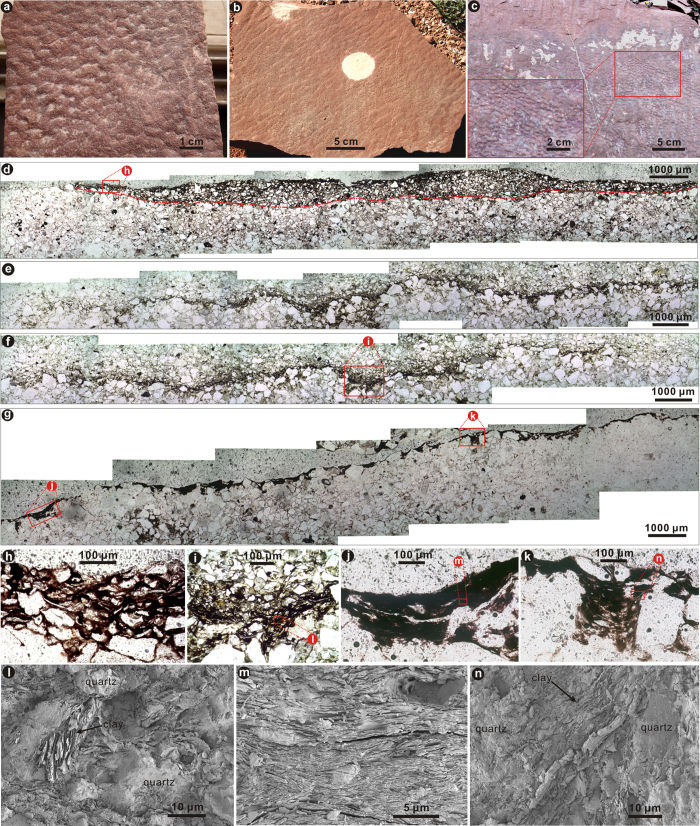
Photographs and thin section photomicrographs of wrinkle structures. (**a**) Cross-cutting wrinkle structures. (**b**) Parallel wavy wrinkle structures. (**c**) Bulge-like wrinkle structure, with elongate to irregular bulges in close-up view (bottom left). (**d**) Thin section of wrinkle features in (**a**). (**e**,**f**) Thin section of the complete structure of the wrinkles. (**g**) Thin section of wrinkle features in Fig. 3a. (**h**-**k**) Close-up views of indicated portions of (**d**), (**f**) and (**g**). (**l**-**n**) SEM images showing the presence of the clay laminae and its detailed internal structure from (**i**-**k**). D.L.C. created this figure using CorelDRAW14.

Four wrinkle structure types were recognized through studies of morphology and microscopic characteristics. Cross cutting wrinkle structures^23^ are preserved as bifurcating and frequently interconnected crests and intervening troughs on upper bedding surfaces of fine sandstones, and one set of wrinkles cuts across another set at a low angle (Fig. 2a). The crests are 2–5 mm in height, 2–6 mm in width, irregular and round-topped. Microscopic features seen in longitudinal thin sections show 0.1–0.4 mm thick, wavy, crinkly, dark and opaque clay laminae on the surfaces of the wrinkle structures (Fig. 2d,h). Fine-grained quartz crystals are abundant in the surface laminae as floating grains and these differ from the underlying grains in size. The clay laminae and the floating quartz grains suggest that the microbial mat trapped sand grains from the surrounding environment, based upon comparative study between ancient and modern microbial mat growth^1^.

Parallel wavy wrinkle structures are preserved as sinuously curved continuous crests, separated by parallel narrow troughs, extending over 15 cm or more on the upper surface of fine sandstone beds (Fig. 2b). The continuous crests of these structures are invariably flat-topped, and the height of individual crests usually ranges from 1 to 2 mm, and their spacing from 10 to 15 mm. Microscopic thin sections perpendicular to the structural trend show that the clay laminae are completely encased in sediment above and below, preserving the complete structure of the wrinkles (Fig. 2e,f,i). SEM images show that the quartz crystals are surrounded by clay laminae (Fig. 2l). This structure is considered to result from mat deformation, perhaps the crumpling of a disrupted and marginally detached mat^24^. Recent wave tank experiments suggest that microbial aggregates can form such wrinkle structures with morphological biosignatures at the sediment-water interface in wave-dominated environments^25^.

Bulge-like wrinkle structures are characterized by patches of elongate to irregular bulges measuring 2–5 mm in length on the upper surfaces of fine-grained sandstones (Fig. 2c). In appearance, bulges with similar geometry and orientation are preserved in discrete patches, presumably retained by covering microbial mats^26^. Preservation of the bulge-like wrinkles and their particular peripheries reflect subsurface morphological features developed beneath a microbial mat^24^, and suggest that at least two generations of microbial development occurred prior to burial.

**Figure 3:**
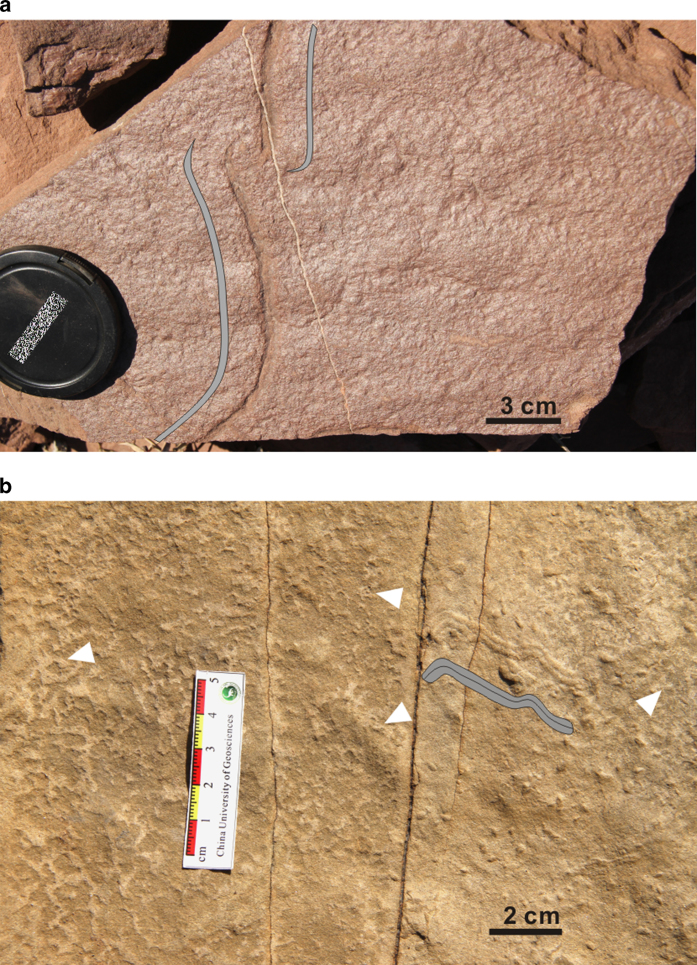
Examples of trace fossils associated with mats. (**a**) Back-filled tunnel cutting through biomat wrinkle structures in the Liujiagou Formation, indicating the trace maker may have mined the microbial mats for food resources. (**b**) Back-filled trace fossil associated with wrinkle structures (arrowed) in the Liujiagou Formation. Grey sketches in (**a**) and (**b**) are offset from the burrows, and indicate their shape and size. D.L.C. created this figure using CorelDRAW14.

“Old-elephant-skin textures”^27^ or briefly “elephant skin”^28^ wrinkle structures were found on the bedding surfaces of the fine-grained sandstones (Fig. 3a). The corrugated surface is characterized by an irregular and geometrically distinct polygonal texture. Individual networks are 3–10 mm in width, with a maximum vertical relief of 5 mm. Dark clay laminae overlying the surface have been observed by microscopic study of thin sections (Fig. 2g,j,k). In addition, SEM images show that the superposed clay laminae wrapped around the quartz grains (Fig. 2m,n). Previous studies suggested that this structure might form when mats provided a veneer between the lithologically identical overlying and underlying beds^2^.

## Discussion

**Figure 4:**
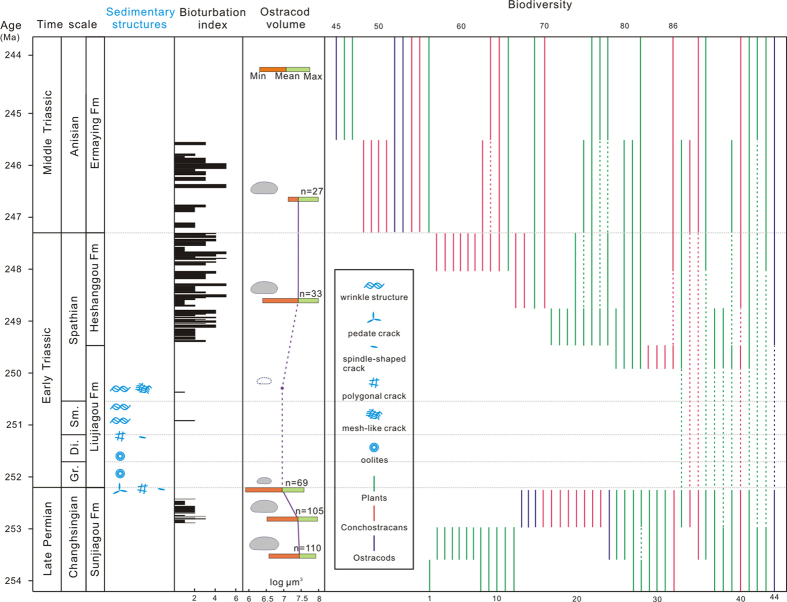
Variations in sedimentary structures, bioturbation index, ostracod volume, plants, ostracods and conchostracans from the Late Permian to Middle Triassic. Wrinkle structures are most common in the Liujiagou Formation, but not present in the Sunjiagou, Heshangou and Ermaying formations. The bioturbation index was measured from the Dayulin section. Fossil data are from Supplementary Tables S1-4. The data on ostracod size is in Supplementary Table S5. Abbreviations: Di., Dienerian; Fm, Formation; Gr., Griesbachian; Ma, million years ago; Sm., Smithian. D.L.C. created this figure using CorelDRAW14.

Blooms of microbial-related sedimentary structures are usually caused by a combination of biotic and sedimentological factors, such as bioturbation, grazing pressure, nutrient levels and sediment dynamics. Modern studies show that the intensity of bioturbation directly determines the presence or absence of microbial mat communities^29^. The geological record suggests that microbial communities usually proliferated in the aftermath of mass extinctions in the post-Cambrian^5^,^7^,^8^,^9^, in both carbonate and siliciclastic settings. As the greatest crisis of the Phanerozoic, current studies show that the land ecosystem suffered marked biotic losses during the P-Tr mass extinction^15^,^16^,^17^. There is strong evidence for the absence of metazoans and of bioturbation in the Liujiagou Formation in North China, and from the varying bioturbation index measured from the Dayulin section through the upper Sunjiagou Formation to the Ermaying Formation (Fig. 4). Decreased grazing pressure, coupled with low-level bioturbation following the P-Tr crisis, promoted the formation and preservation of microbial-related sedimentary structures. The abundant and diverse wrinkle structures in the Early Triassic of North China that we report here suggest the presence of microbe-dominated aquatic ecosystems in terrestrial facies as a response to the environmental crisis following the P-Tr mass extinction.

Nutrient levels are significant in ecosystem construction. Studies of modern fresh waters demonstrate that microbial biomass responds positively to nutrient enrichment, with enhancement of the activities of extracellular enzymes and of metabolic rates^30^. Increased rates of terrestrial weathering have been proved near the P-Tr boundary^31^, which must have resulted in a significant increase in sediment fluxes^32^. Such large increases in sediment flux might have profoundly enhanced nutrient supply to rivers and oceans. Nutrient enrichment would promote the spread of sensitive microbial communities in both terrestrial (this study) and marine depositional systems^7^,^9^.

Physical sedimentary processes also play an important role in the formation of microbial mat-related sedimentary structures, according to the following sequence of events^1^,^33^: (1) binding, in which individual microbes actively assemble to form a microbial mat; (2) biostabilization, in which microbial mats escape destruction by erosion, but are remoulded to a different shape; (3) baffling and trapping, in which the mats accumulate sedimentary particles of a particular size on their surface; and (4) fossilization, in which the mats are buried by rapid pulses of fine-grained sediment deposition, and preserved with atypical features in the rock record. Above all, extrinsic factors, including the size of the sediment and the effects of hydraulics and sediment dynamics, are closely linked with the formation of microbially-related sedimentary structures, among key physical sedimentary processes^33^. The occurrence of the oolitic beds, accompanied by hummocky cross beddings and flat-pebble conglomerates in the Liujiagou Formation in the studied sections, are similar to microbialites and oolites from the Buntsandstein Group in Germany, which are thought to have been deposited in an alkaline playa lake^34^, although other authors argued a marine origin^35^. The extensive occurrences of such sedimentary structures may imply strong water energy and potential geochemical anomalies in continental lakes worldwide, as another sedimentary response to the drastic upheavals in Early Triassic ecosystems and climates^36^.

Oceanic anoxia and lethal warming were thought to have been significant components of selective extinction during the P-Tr marine crisis^37^,^38^, and only those organisms with high tolerance levels to hypoxia and warming could survive in the mid-water refuge zone^37^. In lakes on land, the situation might have been worse. The rate of temperature increase would be greater on land than in the sea. Reduced atmospheric oxygen content^38^, elevated temperature^39^, and a large amount of organic input^32^ would dramatically decrease the solubility of oxygen and lead to hypoxic to anoxic lakes. This situation is in agreement with the finding of much-reduced bioturbation and the lack of body fossils of invertebrates in the early Early Triassic of North China (Fig. 4). Furthermore, our investigation of size variation in the terrestrial ostracod *Darwinula* from the Late Permian to Middle Triassic shows a sharp decrease during the P-Tr crisis (Fig. 4). The Lilliput effect^40^, well known among Early Triassic marine invertebrates^41^, also occurred among aquatic invertebrates on land, implying similarly stressed environments.

Finally, we identify evidence that certain lake-dwellers exploited the microbial mats as oases of oxygen and nutrients. This is based on the occurrence of rare trace fossils in the Liujiagou Formation, running parallel to the surface of the microbial mats (Fig. 3). The burrow cutting through the biomat might indicate that the trace maker may have mined the microbial mats for food resources (Fig. 3a). This evidence suggests that trace-making animals inhabited settings with microbial populations, and may have mined microbially bound sediments for food resources, a phenomenon that has been mentioned in the study of the relationship between the earliest mobile animals and settings with high microbial populations in the Precambrian^26^,^42^. A modern analogue is a warm, hypoxic, hypersaline lagoon in Venezuela in which it was found^43^ that O_2_ levels in the cyanobacterial, photosynthesizing microbial mats are four times higher than in the overlying hypoxic water column, and so these provide a suitable environment for animals to exploit biomat oxygen sources whilst foraging for food. Here, we hypothesize that survivors of the P-Tr mass extinction might have evolved in similar environments during the Early Triassic, effectively exploiting the abundant microbial mats, which acted as oases rich in oxygen and food within otherwise poorly oxygenated settings.

## Methods

Sedimentary structures were recorded in detail by field observations in the three studied sections. A total of 133 samples were collected. All the features were photographed with a Canon EOS 7D digital camera. Cut slabs of rock and thin sections were produced using standard techniques. Sixty-five thin sections were made, and these were examined and photographed using a LEICA-DM-750P microscope. Three thin sections were employed for ultramicroscopic studies under a HITACHI-SU8010 SEM with a iXRF EDS.

## Additional Information

**How to cite this article**: Chu, D. *et al.* Early Triassic wrinkle structures on land: stressed environments and oases for life. *Sci. Rep.* **5**, 10109; doi: 10.1038/srep10109 (2015).

## Supplementary Material

Supplementary Information
