# Supplementary material for: Early Triassic wrinkle structures on land: stressed environments and oases for life
Source: Sci Rep. 2015 Jun 9;5:10109. doi: 10.1038/srep10109 (PMC4460569; doi:10.1038/srep10109)
Supplement: Supplementary Information [file srep10109-s1.pdf]

Supplementary material

Early Triassic wrinkle structures on land: stressed environments and oases for life

Daoliang Chu[1], Jinnan Tong[1*], Haijun Song[1*], Michael J. Benton[2], David J. Bottjer[3], Huyue Song[1] and Li Tian[1]

[1]State Key Laboratory of Biogeology and Environmental Geology, China University of Geosciences, Wuhan 430074, China

[2]School of Earth Sciences, University of Bristol, Bristol, BS8 1RJ, UK

[3]Department of Earth Sciences, University of Southern California, Los Angeles, CA 90089, USA.

*Corresponding authors: jntong@cug.edu.cn (J.N.T.); haijun.song@aliyun.com (H.J.S)

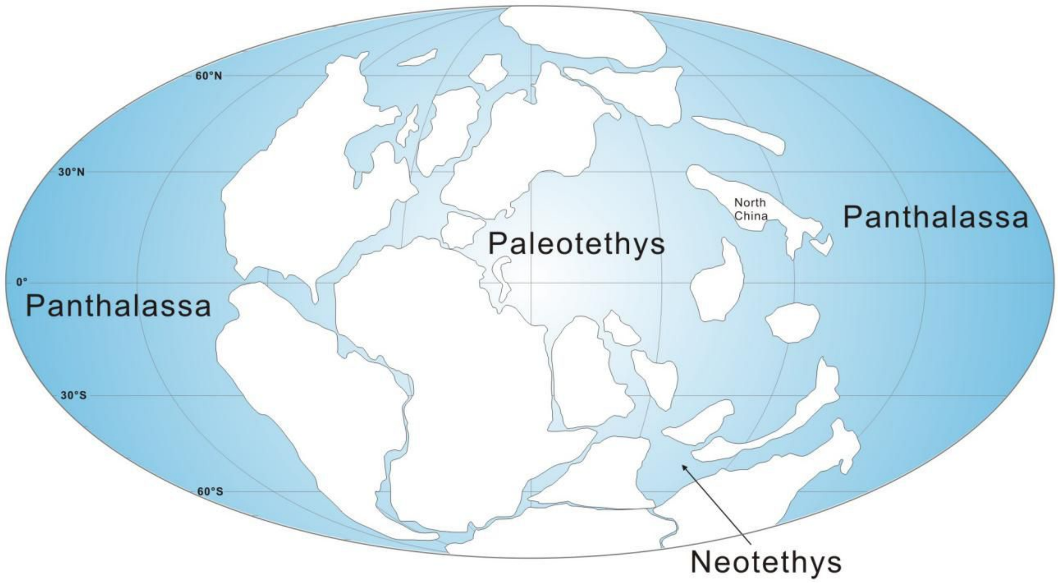

Figure S1. **Paleogeography map of the Permian-Triassic boundary interval** (modified from Erwin, D. H., 1994, The Permo-Triassic extinction: Nature 367, 231-236. doi:10.1038/367231a0. Figure 1). D.L.C. modified this figure after Erwin (1994) using CorelDRAW14.

The copyright permission:

http://s100.copyright.com/CustomerAdmin/PLF.jsp?ref=357e403a-2d57-4144-896a-9cba2e3efa6b

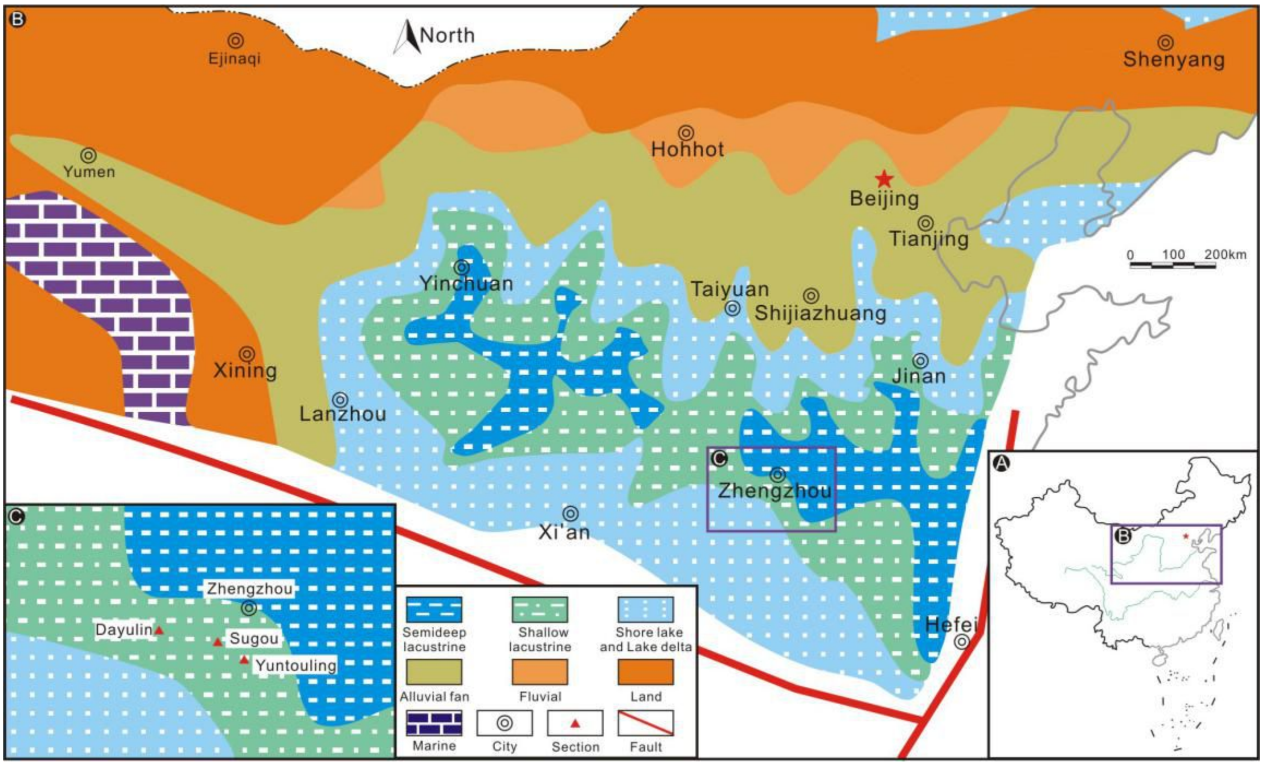

Figure S2. **Latest Permian paleogeographic map of North China** (modified from Zhu, R.K., Deng, S.H., Xu, H.X., and Guo, H.L., 2007, Lithofacies palaeogeography of the Permian in northern China: Journal of Palaeogeography 9, 133-142. Figure 3). A: Territory of China; B: Lithofacies paleogeography of the Latest Permian in Northern China; C: The red triangles show the location of studied sections: Dayulin, Sugou and Yuntouling sections. D.L.C. modified this figure after Zhu et al. (2007) using CorelDRAW14.

The copyright permission: the PDF file of the copyright permission from the publisher.

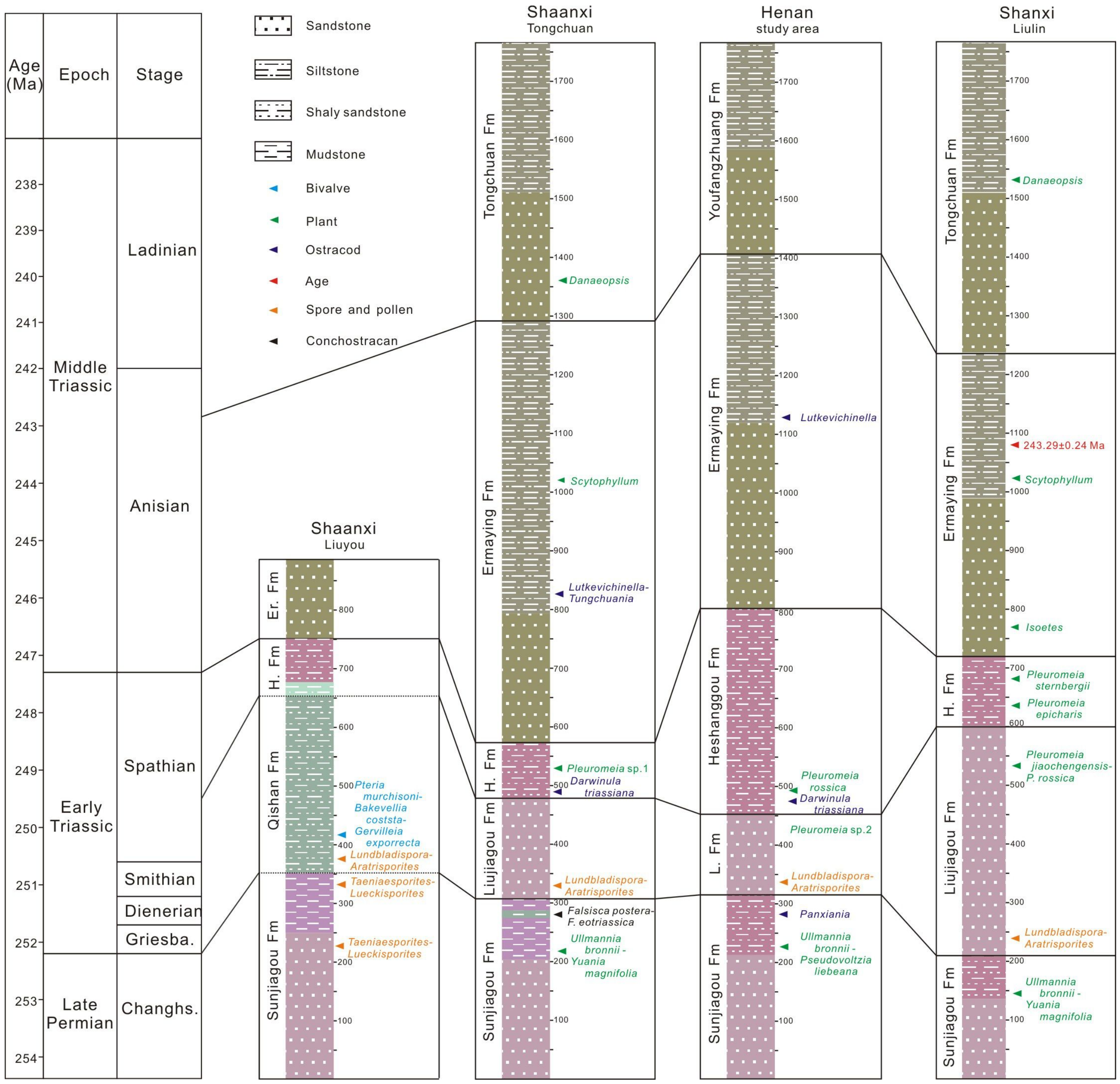

Figure S3. **Biostratigraphic and lithostratigraphic sequences from Late Permian to Middle Triassic in different areas of North China.** D.L.C. created this figure using CorelDRAW14.

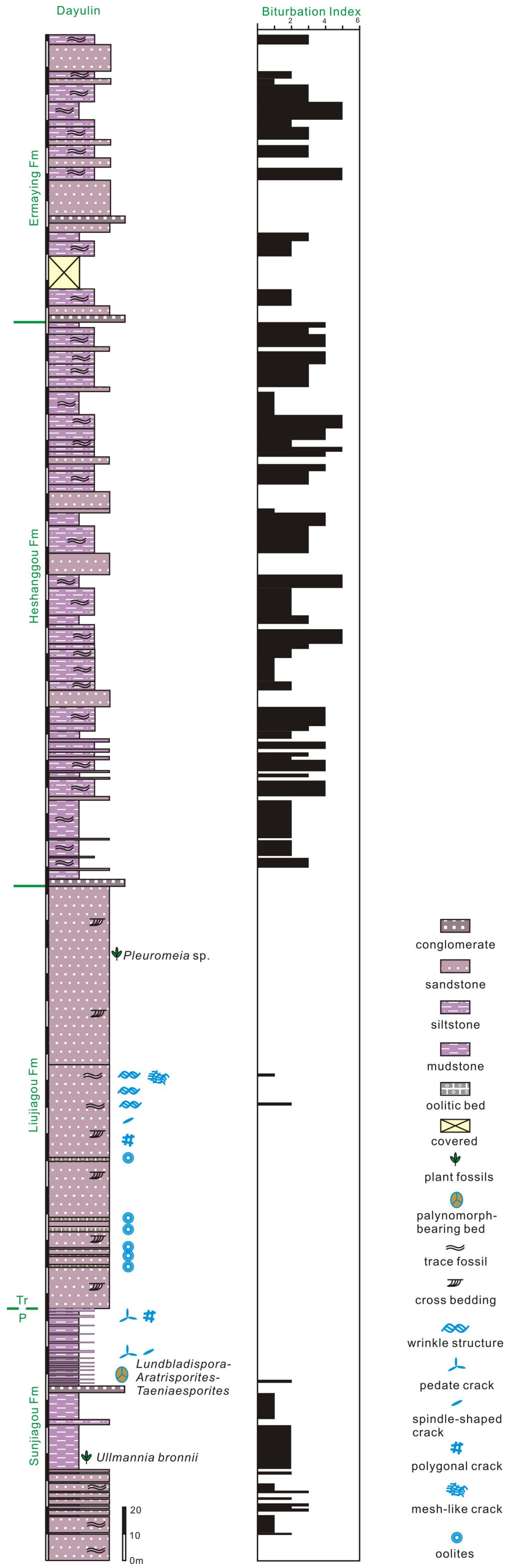

Figure S4. **Bioturbation index of Dayulin section.** D.L.C. created this figure using CorelDRAW14.

Table S1. **Fossil list in the Figure 4.**

No.	Fossil	Genus	No.	Fossil	Genus
1	Plant	*Pityospermum*	29	Plant	*Sphenophyllum*
2	Plant	*Algites*	30	Plant	*Callipteris*
3	Plant	*Gaussia*	31	Plant	*Yuania*
4	Plant	*Squarmacarpus*	32	Conchostracan	*Estherina*
5	Plant	*Carpolithus*	33	Plant	*Peltaspermum*
6	Plant	*Lesleya*	34	Conchostracan	*Sphaerestheria*
7	Plant	*Esterella*	35	Conchostracan	*Euestheria*
8	Plant	*Discinites*	36	Plant	*Scytophyllum*
9	Plant	*Asterophyllites*	37	Plant	*Pecopteris*
10	Plant	*Carpolithus*	38	Plant	*Phyllotheca*
11	Plant	*Norinia*	39	Plant	*Taeniopteris*
12	Plant	*Walchia*	40	Conchostracan	*Palaeolimnadia*
13	Ostracod	*Iniella*	41	Plant	*Calamites*
14	Ostracod	*Tomiella*	42	Plant	*Sphenobaiera*
15	Ostracod	*Kemeroviana*	43	Plant	*Cladophlebis*
16	Conchostracan	*Palaeolimnadiopsis*	44	Ostracod	*Darwinula*
17	Conchostracan	*Falsisca*	45	Ostracod	*Tungchuanina*
18	Conchostracan	*Huangheetheria*	46	Plant	*Danaeopsis-Bernouillia*
19	Conchostracan	*Hemicycloleaia*	47	Plant	*Ctenozamites*
20	Conchostracan	*Rostroleaia*	48	Conchostracan	*Leptolimnadia*
21	Conchostracan	*Costestheria*	49	Conchostracan	*Paleoleptestheria*
22	Conchostracan	*Pemphicyclus*	50	Conchostracan	*Loxomegaglypta*
23	Conchostracan	*Rhyssestheria*	51	Conchostracan	*Lioestheria*
24	Ostracod	*Panxiania*	52	Ostracod	*Lutkevichinella*
25	Plant	*Pseudovoltzia*	53	Ostracod	*Shansinella*
26	Plant	*Tatarina*	54	Conchostracan	*Xiangxiella*
27	Plant	*Quadrocladus*	55	Conchostracan	*Protomonocarina*
28	Plant	*Ullmannia*	56	Plant	*Nilssonia*

57	Conchostracan	*Punctestheria*			
58	Conchostracan	*Trisitum*			
59	Conchostracan	*Brachystheria*			
60	Conchostracan	*Anyuanestheria*			
61	Conchostracan	*Glyptoasmussia*			
62	Conchostracan	*Dictyostriaca*			
63	Conchostracan	*Loxomegaglypta*			
64	Conchostracan	*Leptonemia*			
65	Conchostracan	*Eosolimnadia*			
66	Plant	*Equisetites*			
67	Conchostracan	*Triasestheria*			
68	Conchostracan	*Polygrapta*			
69	Plant	*Lycostrobus-Isoetes*			
70	Conchostracan	*Aquilonoglypta*			
71	Plant	*Schizoneura*			
72	Plant	*Glossotheca-Paleovittaria*			
73	Plant	*Otozamites*			
74	Plant	*Neuropteridium*			
75	Plant	*Yuccites-"Gangamopteris"*			
76	Plant	*Tongchuanophyllum*			
77	Plant	*Neoglossopteris-"Glossopteris"*			
78	Plant	*Glossophyllum*			
79	Plant	*Willsiostrobus*			
80	Plant	*Pleuromeia*			
81	Plant	*Anomopteris-Crematopteris*			
82	Plant	*Voltzia*			
83	Conchostracan	*Diaplexa*			
84	Conchostracan	*Liolimnadia*			
85	Conchostracan	*Cornia*			
86	Conchostracan	*Gabonestheria*			

Table S2. **Plant fossils data from Late Permian to Middle Triassic in North China.**

	Time	Species	Formation- Location	Reference
Plant	Late Changhsingian	*Ullmannia bronnii*	Lower-middle Upper Sunjiagou Fm- Liulin, Shanxi; Linxian, Shanxi; Yiyang, Henan	Wang and Wang, 1986
Plant	Late Changhsingian	*Ullmannia frumentaria*	middle Upper Sunjiagou Fm- Liulin, Shanxi	Wang and Wang, 1986
Plant	Late Changhsingian	*Yuania magnifolia*	Middle Sunjiagou Fm- Liulin, Shanxi	Wang and Wang, 1986
Plant	Late Changhsingian	*Algites junduensis*	Middle Sunjiagou Fm- Liulin, Shanxi	Wang and Wang, 1986
Plant	Late Changhsingian	*Sphenophyllum* sp.1	Middle Sunjiagou Fm- Liulin, Shanxi	Wang and Wang, 1986
Plant	Late Changhsingian	*Sphenophyllum* sp.2	Middle Sunjiagou Fm-Liulin, Shanxi	Wang and Wang, 1986
Plant	Late Changhsingian	*Sphenophyllum* spp.	Middle Sunjiagou Fm- Liulin, Shanxi	Wang and Wang, 1986
Plant	Late Changhsingian	*Calamites* sp.	Middle Sunjiagou Fm- Liulin, Shanxi	Wang and Wang, 1986
Plant	Late Changhsingian	*Asterophyllites equisetofomis*	middle Upper Sunjiagou Fm- Liulin, Shanxi; Jiyuan, Henan	Wang and Wang, 1986
Plant	Late Changhsingian	*Phyllotheca* sp.	Middle Sunjiagou Fm- Liulin, Shanxi	Wang and Wang, 1986
Plant	Late Changhsingian	*Discinites sunjiagouensis*	Middle Sunjiagou Fm- Liulin, Shanxi	Wang and Wang, 1986
Plant	Late Changhsingian	*Discinites fimbriata*	Middle Sunjiagou Fm- Liulin, Shanxi	Wang and Wang, 1986
Plant	Late Changhsingian	*Sphenopteris* spp.	Middle Sunjiagou Fm- Liulin, Shanxi	Wang and Wang, 1986
Plant	Late Changhsingian	*Pecopteris* cf. *arcuata*	Middle Sunjiagou Fm- Liulin, Shanxi	Wang and Wang, 1986
Plant	Late Changhsingian	*Pecopteris* spp.	Middle Sunjiagou Fm- Liulin, Shanxi	Wang and Wang, 1986
Plant	Late Changhsingian	*Cladophlebis* sp.	Middle Sunjiagou Fm- Liulin, Shanxi	Wang and Wang, 1986
Plant	Late Changhsingian	*Callipteris lobulata*	Middle Sunjiagou Fm- Liulin, Shanxi	Wang and Wang, 1986
Plant	Late Changhsingian	*Callipteris martinsii*	Middle Sunjiagou Fm- Liulin, Shanxi	Wang and Wang, 1986
Plant	Late Changhsingian	*Callipteris papillosa*	Middle Sunjiagou Fm- Liulin, Shanxi	Wang and Wang, 1986
Plant	Late Changhsingian	*Scytophyllum sunjiagouensis*	Middle Sunjiagou Fm- Liulin, Shanxi	Wang and Wang, 1986
Plant	Late Changhsingian	*Tatarina* cf. *sinuosa*	Middle Sunjiagou Fm- Liulin, Shanxi	Wang and Wang, 1986

Plant	Late Changhsingian	*Tatarina mirabilis*	Middle Sunjiagou Fm- Liulin, Shanxi	Wang and Wang, 1986
Plant	Late Changhsingian	*Peltaspermum dafengshanensis*	Middle Sunjiagou Fm- Liulin, Shanxi	Wang and Wang, 1986
Plant	Late Changhsingian	*Phylladoderma* (*Aequistomia*) cf. *aequalis*	Middle Sunjiagou Fm- Liulin, Shanxi	Wang and Wang, 1986
Plant	Late Changhsingian	*Gaussia shanxiensis*	Middle Sunjiagou Fm- Liulin, Shanxi	Wang and Wang, 1986
Plant	Late Changhsingian	*Sphenobaiera micronervis*	Middle Sunjiagou Fm- Liulin, Shanxi; Linxian, Shanx	Wang and Wang, 1986
Plant	Late Changhsingian	*Walchia* sp.	Middle Sunjiagou Fm- Liulin, Shanxi	Wang and Wang, 1986
Plant	Late Changhsingian	*Pseudovoltzia liebeana*	Middle Sunjiagou Fm- Liulin, Shanxi	Wang and Wang, 1986
Plant	Late Changhsingian	*Quadrocladus solmsii*	Middle Sunjiagou Fm- Liulin, Shanxi	Wang and Wang, 1986
Plant	Late Changhsingian	*Quadrocladus heterodermus*	Middle Sunjiagou Fm- Liulin, Shanxi	Wang and Wang, 1986
Plant	Late Changhsingian	*Quadrocladus* sp.	Middle Sunjiagou Fm- Linxian, Shanxi	Wang and Wang, 1986
Plant	Late Changhsingian	*Pityospermum dafengshanensis*	middle Lower Sunjiagou Fm- Liulin, Shanxi; Linxian, Shanxi; Yiyang, Henan	Wang and Wang, 1986
Plant	Late Changhsingian	*Esterella* sp.	Middle Sunjiagou Fm- Liulin, Shanxi	Wang and Wang, 1986
Plant	Late Changhsingian	*Lesleya anastomosis*	Middle Sunjiagou Fm- Liulin, Shanxi	Wang and Wang, 1986
Plant	Late Changhsingian	*Norinia* sp.	Middle Sunjiagou Fm- Liulin, Shanxi	Wang and Wang, 1986
Plant	Late Changhsingian	*Taeniopteris liulinensis*	Middle Sunjiagou Fm- Liulin, Shanxi	Wang and Wang, 1986
Plant	Late Changhsingian	*Taeniopteris longifolia*	Middle Sunjiagou Fm- Liulin, Shanxi	Wang and Wang, 1986
Plant	Late Changhsingian	*Taeniopteris nystroemii*	Middle Sunjiagou Fm- Liulin, Shanxi	Wang and Wang, 1986
Plant	Late Changhsingian	*Taeniopteris* cf. *serrulata*	Middle Sunjiagou Fm- Liulin, Shanxi	Wang and Wang, 1986
Plant	Late Changhsingian	*Taeniopteris taiyuanensis*	Middle Sunjiagou Fm- Liulin, Shanxi	Wang and Wang, 1986
Plant	Late Changhsingian	*Cardiocarpus* cf. *triangularis*	Middle Sunjiagou Fm- Liulin, Shanxi	Wang and Wang, 1986
Plant	Late Changhsingian	*Carpolithus* sp.	Middle Sunjiagou Fm- Liulin, Shanxi	Wang and Wang, 1986
Plant	Late Changhsingian	*Squarmacarpus cuneiformus*	Middle Sunjiagou Fm- Liulin, Shanxi	Wang and Wang, 1986

Plant	Early Spathian	*Pleuromeia rossica*	Upper Liujiagou Fm- Yushe, Shanxi; Jiaocheng, Shanxi	Zhou and Zhou, 1983
Plant	Early Spathian	*Pleuromeia jiaochengensis*	Upper Liujiagou Fm- Jiaocheng, Shanxi	Zhou and Zhou, 1983
Plant	Early Spathian	*Neocalamites* sp.	Upper Liujiagou Fm- Jiaocheng, Shanxi	Zhou and Zhou, 1983
Plant	Early Spathian	*Phyllotheca yusheensis*	Upper Liujiagou Fm- Yushe, Shanxi	Zhou and Zhou, 1983
Plant	Early Spathian	*Phyllotheca yusheensis*	Upper Liujiagou Fm- Jiaocheng, Shanxi	Zhou and Zhou, 1983
Plant	Early Spathian	*Crematopteris* sp.	Upper Liujiagou Fm- Jiaocheng, Shanxi	Zhou and Zhou, 1983
Plant	Early Spathian	*Scyhophyllum* sp.	Upper Liujiagou Fm- Yushe, Shanxi	Zhou and Zhou, 1983
Plant	Early Spathian	*Yuccites* sp.	Upper Liujiagou Fm- Yushe, Shanxi	Zhou and Zhou, 1983
Plant	Early Spathian	*Masculostrobus* cf. *acuminatus*	Upper Liujiagou Fm- Yushe, Shanxi	Zhou and Zhou, 1983
Plant	Early Spathian	*Neoglossopteris shanxiensis*	Upper Liujiagou Fm- Yushe, Shanxi	Zhou and Zhou, 1983
Plant	Early Spathian	*Gangamopteris qinshuiensis*	Upper Liujiagou Fm- Yushe, Shanxi	Zhou and Zhou, 1983
Plant	Early Spathian	*Botrychiopsis* (*Gondwanidium*) sp.	Upper Liujiagou Fm- Yushe, Shanxi	Zhou and Zhou, 1983
Plant	Early Spathian	*Palaeovittaria* sp.	Upper Liujiagou Fm- Yushe, Shanxi	Zhou and Zhou, 1983
Plant	Early Spathian	*Eretmonia* sp.	Upper Liujiagou Fm- Yushe, Shanxi	Zhou and Zhou, 1983
Plant	Early Spathian	*Samaropsis milleri*	Upper Liujiagou Fm- Yushe, Shanxi	Zhou and Zhou, 1983
Plant	Middle Spathian	*Pleuromeia epicharis*	Lower Heshanggou Fm- Heshun, Shanxi; Yushe, Shanxi; Yima, Henan	Wang and Wang, 1990a
Plant	Middle Spathian- Late Spathian	*Pleuromeia sternbergii*	Heshanggou Fm- Heshun, Shanxi; Yushe, Shanxi; Shouyang, Shanxi; Pingyao, Shanxi; Puxian, Shanxi; Jiyuan, Henan; FengFeng, Hebei; Chengde, Hebei	Wang and Wang, 1990a
Plant	Middle Spathian	*Pleuromeia rossica*	Bottom of Heshanggou Fm- Yushe, Shanxi; Jiaocheng, Shanxi	Wang and Wang, 1990a
Plant	Middle Spathian	*Isoetites sagittatus*	Lower Heshanggou Fm- Puxian, Shanxi	Wang and Wang, 1990a
Plant	Late Spathian	*Annalepis* sp.	Upper Heshanggou Fm- Puxian, Shanxi	Wang and Wang, 1990a

Plant	Middle Spathian	*Mesolepidodendron xiabanchengensis*	Lower Heshanggou Fm- Jiyuan, Henan	Wang and Wang, 1990a
Plant	Middle Spathian	*Sphenophyllum* sp.	Bottom of Heshanggou Fm- Yushe, Shanxi	Wang and Wang, 1990a
Plant	Middle Spathian- Late Spathian	*Calamites shanxiensis*	Heshanggou Fm- Yushe, Shanxi;Shilou, Shanxi;Heshun, Shanxi	Wang and Wang, 1990a
Plant	Middle Spathian	*Lobatannufaria* sp.	Bottom of Heshanggou Fm- Yushe, Shanxi	Wang and Wang, 1990a
Plant	Middle Spathian- Late Spathian	*Macrostachya gracilis*	Lower-Middle Heshanggou Fm- Heshun, Shanxi	Wang and Wang, 1990a
Plant	Middle Spathian	*Phyllotheca bicruris*	Bottom of Heshanggou Fm- Yushe, Shanxi	Wang and Wang, 1990a
Plant	Middle Spathian-	*Phyllotheca yusheensis*	Lower Heshanggou Fm- Yushe, Shanxi; Shouyang, Shanxi	Wang and Wang, 1990a
Plant	Middle Spathian	*Phyllotheca* sp.	Bottom of Heshanggou Fm- Yushe, Shanxi	Wang and Wang, 1990a
Plant	Late Spathian	*Neocalamites* sp.	Upper Heshanggou Fm- Puxian, Shanxi	Wang and Wang, 1990a
Plant	Middle Spathian	*Equisetites* sp.	Lower Heshanggou Fm- Yushe, Shanxi	Wang and Wang, 1990a
Plant	Middle Spathian	*Schizoneura* (*Eehinostachys*) *megaphylla*	Lower Heshanggou Fm- Yushe, Shanxi; Heshun, Shanxi	Wang and Wang, 1990a
Plant	Middle Spathian	*Anomopteris minima*	Lower Heshanggou Fm- Shouyang, Shanxi	Wang and Wang, 1990a
Plant	Late Spathian	*Anomopteris* cf. *mougeotii*	Upper Heshanggou Fm- Yiyang, Henan	Wang and Wang, 1990a
Plant	Middle Spathian	*Crematopteris* cf. *typic*	Lower Heshanggou Fm- Puxian, Shanxi; Pingyao, Shanxi; Jiyuan, Henan	Wang and Wang, 1990a
Plant	Late Spathian	*Crematopteris* sp.	Bottom of Heshanggou Fm- Yushe, Shanxi	Wang and Wang, 1990a
Plant	Late Spathian	*Neuropteridium curvinerve*	Bottom of Heshanggou Fm- Yushe, Shanxi	Wang and Wang, 1990a
Plant	Middle Spathian- Late Spathian	*Neuropteridium* sp.	Heshanggou Fm- Puxian, Shanxi; Yiyang, Henan	Wang and Wang, 1990a
Plant	Middle Spathian	*Ctadophlebis* sp.1	Bottom of Heshanggou Fm- Yushe, Shanxi	Wang and Wang, 1990a
Plant	Middle Spathian	*Ctadophlebis* sp.2	Bottom of Heshanggou Fm- Yushe, Shanxi	Wang and Wang, 1990a
Plant	Middle Spathian	*sphenopteris yusheensis*	Bottom of Heshanggou Fm- Yushe, Shanxi	Wang and Wang, 1990a
Plant	Middle Spathian	*Sphenopterisdelabens*	Bottom of Heshanggou Fm- Yushe, Shanxi	Wang and Wang, 1990a
Plant	Middle Spathian- Late Spathian	*Sphenopteris* sp.	Middle Heshanggou Fm- Heshun, Shanxi	Wang and Wang, 1990a

Plant	Middle Spathian- Late Spathian	*Glossophylhan* sp.	Heshanggou Fm- Yushe, Shanxi; Yiyang, Henan	Wang and Wang, 1990a
Plant	Middle Spathian	*Peltaspermum lobulatum*	Bottom of Heshanggou Fm- Yushe, Shanxi	Wang and Wang, 1990a
Plant	Middle Spathian- Late Spathian	*Peltaspermum calycmum*	Lower-Middle Heshanggou Fm- Heshun,Shanxi; Yima, Henan	Wang and Wang, 1990a
Plant	Late Spathian	*Scytophyllum* cf. *bergeri*	Upper Heshanggou Fm- Yiyang, Henan	Wang and Wang, 1990a
Plant	Middle Spathian	*Thinnfeldia monopinnata*	Bottom of Heshanggou Fm- Yushe, Shanxi	Wang and Wang, 1990a
Plant	Middle Spathian	*Tongchuanophyllum minimum*	Bottom of Heshanggou Fm- Yushe, Shanxi;Shouyang, Shanxi	Wang and Wang, 1990a
Plant	Middle Spathian- Late Spathian	*Tongchuanopyllum* cf. *concinnum*	Lower-Middle Heshanggou Fm- Yushe, Shanxi	Wang and Wang, 1990a
Plant	Middle Spathian	*Tongchuanopttum* cf. *mshensiense*	Lower Heshanggou Fm- Yushe, Shanxi	Wang and Wang, 1990a
Plant	Middle Spathian	*Glossopteris shanxiensis*	Bottom of Heshanggou Fm- Yushe, Shanxi	Wang and Wang, 1990a
Plant	Middle Spathian- Late Spathian	*Gangamopteris qinshuiensis*	Bottom of Heshanggou Fm- Yushe, Shanxi	Wang and Wang, 1990a
Plant	Middle Spathian	*Gangamopteris tuncunensis*	Bottom of Heshanggou Fm- Yushe, Shanxi	Wang and Wang, 1990a
Plant	Middle Spathian	*Zamiopteris minor*	Bottom of Heshanggou Fm- Yushe, Shanxi	Wang and Wang, 1990a
Plant	Middle Spathian	*Gtossotheca petiolata*	Bottom of Heshanggou Fm- Yushe, Shanxi	Wang and Wang, 1990a
Plant	Middle Spathian	*Gtossotheca cuneiformis*	Bottom of Heshanggou Fm- Yushe, Shanxi	Wang and Wang, 1990a
Plant	Middle Spathian	*Gtossotheca cochlearis*	Bottom of Heshanggou Fm- Yushe, Shanxi	Wang and Wang, 1990a
Plant	Middle Spathian	*Euryphyttum* sp.	Bottom of Heshanggou Fm- Yushe, Shanxi	Wang and Wang, 1990a
Plant	Middle Spathian	*Pateovittaria shanxiensis*	Bottom of Heshanggou Fm- Yushe, Shanxi	Wang and Wang, 1990a
Plant	Middle Spathian	*Otozamites* sp.	Bottom of Heshanggou Fm- Yushe, Shanxi	Wang and Wang, 1990a
Plant	Middle Spathian	*Williamsonia lanceolobata*	Bottom of Heshanggou Fm- Yushe, Shanxi	Wang and Wang, 1990a
Plant	Middle Spathian	*Ruehleostachys hongyatouensis*	Lower Heshanggou Fm- Yushe, Shanxi;Heshun, Shanxi	Wang and Wang, 1990a
Plant	Middle Spathian	*Voltzia quinquepetala*	Lower Heshanggou Fm- Yushe, Shanxi;Heshun, Shanxi	Wang and Wang, 1990a

Plant	Middle Spathian- Late Spathian	*Voltzia* cf. *heterophylla*	Heshanggou Fm- Yushe, Shanxi;Puxian, Shanxi; Yiyang, Henan	Wang and Wang, 1990a
Plant	Middle Spathian	*Willsiostrobus ligulatus*	Lower Heshanggou Fm- Yushe, Shanxi; Heshun, Shanxi	Wang and Wang, 1990a
Plant	Middle Spathian- Late Spathian	*Willsiostrobus cordiformis*	middle Lower Heshanggou Fm- Yushe, Shanxi; Heshun, Shanxi	Wang and Wang, 1990a
Plant	Middle Spathian	*Willsiostrobus* cf. *denticulatus*	Lower Heshanggou Fm- Heshun, Shanxi; Puxian, Shanxi	Wang and Wang, 1990a
Plant	Middle Spathian	*Yuccites anastomosis*	Lower Heshanggou Fm- Yushe, Shanxi; Heshun, Shanxi; Puxian, Shanxi	Wang and Wang, 1990a
Plant	Middle Spathian- Late Spathian	*Tricranolepis obtusiloba*	Bottom of Heshanggou Fm- Yushe, Shanxi	Wang and Wang, 1990a
Plant	Middle Spathian	*Tricrananthus sagittatus*	Bottom of Heshanggou Fm- Yushe, Shanxi	Wang and Wang, 1990a
Plant	Middle Spathian	*Tricrananthus lobatus*	Lower Heshanggou Fm- Puxian, Shanxi	Wang and Wang, 1990a
Plant	Middle Spathian	*Cardiocarpus yuccinoides*	Lower Heshanggou Fm- Yushe, Shanxi; Heshun, Shanxi	Wang and Wang, 1990a
Plant	Middle Spathian	*Samaropsis* sp.	Lower Heshanggou Fm- Puxian, Shanxi	Wang and Wang, 1990a
Plant	Middle Spathian	*Corticous impression*	Bottom of Heshanggou Fm- Yushe, Shanxi	Wang and Wang, 1990a
Plant	Early Ansian	*Pleuromeia* sp.	Bottom of Ermaying Fm- Qinxian, Shanxi	Wang and Wang, 1990b
Plant	Early Ansian	*Isoetes ermayingenss*	Bottom of Ermaying Fm- Qinxian, Shanxi; Wupu, Shaanxi	Wang and Wang, 1990b
Plant	Early Ansian	*Lycostrobus petiolatus*	Bottom of Ermaying Fm- Wuxiang, Shanxi	Wang and Wang, 1990b
Plant	Early Ansian	*Calamites shanxiensis*	Bottom of Ermaying Fm- Qinxian, Shanxi;Pingyao,	Wang and Wang, 1990b
Plant	Early Ansian	*Anomopteris ermayingensis*	Bottom of Ermaying Fm- Qinxian, Shanxi	Wang and Wang, 1990b
Plant	Early Ansian	*Lesangeana qinxianensis*	Bottom of Ermaying Fm- Wuxiang, Shanxi	Wang and Wang, 1990b
Plant	Early Ansian	*Lesangeana vogesiaca*	Bottom of Ermaying Fm- Wuxiang, Shanxi	Wang and Wang, 1990b
Plant	Early Ansian	*Lepidopteris* cf. *toretziensis*	Bottom of Ermaying Fm- Qinxian，Shanxi	Wang and Wang, 1990b
Plant	Early Ansian	*Peltaspermum* cf. *rotula*	Bottom of Ermaying Fm- Shanlou, Shanxi	Wang and Wang, 1990b
Plant	Early Ansian	*Peltaspermum* sp.	Bottom of Ermaying Fm- Wuxiang, Shanxi	Wang and Wang, 1990b

Plant	Early Ansian	*Scytophyllum cryptonerve*	Bottom of Ermaying Fm-Ningwu, Shanxi	Wang and Wang, 1990b
Plant	Early Ansian	*Scytophyllum* sp.	Bottom of Ermaying Fm-Qinxian, Shanxi	Wang and Wang, 1990b
Plant	Early Ansian	*Tongchuanophyllum magnifolius*	Bottom of Ermaying Fm-Qinxian, Shanxi; Ningwu, Shanxi	Wang and Wang, 1990b
Plant	Early Ansian	*Nilssonia* sp.	Bottom of Ermaying Fm-Qinxian, Shanxi; Ningwu, Shanxi; Wuxiang, Shanxi	Wang and Wang, 1990b
Plant	Early Ansian	*Voltzia* sp.	Bottom of Ermaying Fm-Wuxiang, Shanxi	Wang and Wang, 1990b
Plant	Early Ansian	*Yuccites* sp.	Bottom of Ermaying Fm-Ningwu, Shanxi	Wang and Wang, 1990b

Table S3. **Ostracode fossils data from Latest Permian to Middle Triassic in North China.**

	Time	Species	Formation- Location	Reference
Ostracode	Latest Changhsingian	*Darwinula aclinis*	Upper Sunjiagou Fm-Ruzhou, Henan	Wang R, 1997
Ostracode	Latest Changhsingian	*Darwinula attenta*	Upper Sunjiagou Fm-Ruzhou, Henan	Wang R, 1997
Ostracode	Latest Changhsingian	*Darwinula* sp.1	Upper Sunjiagou Fm-Ruzhou, Henan	Wang R, 1997
Ostracode	Latest Changhsingian	*Darwinula encelada*	Upper Sunjiagou Fm-Ruzhou, Henan	Wang R, 1997
Ostracode	Latest Changhsingian	*Iniella* spp.	Upper Sunjiagou Fm-Ruzhou, Henan	Wang R, 1997
Ostracode	Latest Changhsingian	*Tomiella borisockaensis*	Upper Sunjiagou Fm-Ruzhou, Henan	Wang R, 1997
Ostracode	Latest Changhsingian	*Kemeroviana argelata*	Upper Sunjiagou Fm-Ruzhou, Henan	Wang R, 1997
Ostracode	Latest Changhsingian	*Panxiania* sp.	Upper Sunjiagou Fm-Yongcheng, Henan	Wang B, 1988
Ostracode	Latest Changhsingian	*Darwinula accepta*	Upper Sunjiagou Fm-Yongcheng, Henan	Wang B, 1988
Ostracode	Latest Changhsingian	*Darwinula ingrata*	Upper Sunjiagou Fm-Yongcheng, Henan	Wang B, 1988
Ostracode	Latest Changhsingian	*Darwinula adducta*	Upper Sunjiagou Fm-Yongcheng, Henan	Wang B, 1988
Ostracode	Latest Changhsingian	*Darwinula auepta*	Upper Sunjiagou Fm-Yongcheng, Henan	Wang B, 1988
Ostracode	Latest Changhsingian	*Darwinula angusia*	Upper Sunjiagou Fm-Yongcheng, Henan	Wang B, 1988
Ostracode	Middle Spathian- Late Spathian	*Darwinula triassiana*	Lower and Middle Heshanggou Fm-Yima, Henan; Upper Heshanggou Fm-Yaoxian, Shaanxi	Pang, 1993；Pang,1989
Ostracode	Middle Spathian- Late Spathian	*Darwinula fengfengensis*	Midle Heshanggou Fm-Yima, Henan	Pang, 1993；Pang,1989
Ostracode	Middle Spathian- Late Spathian	*Darwinula rotundata*	Lower-Middle Heshanggou Fm-Yima, Henan; Upper Heshanggou Fm-Yaoxian, Shaanxi	Pang, 1993；Pang,1989
Ostracode	Middle Spathian- Late Spathian	*Darwinula pseudoinornata*	Middle Heshanggou Fm-Yima, Henan	Pang, 1993；Pang,1989
Ostracode	Middle Spathian- Late Spathian	*Darwinula parva*	Middle Heshanggou Fm-Fugu, Shanxi	Pang, 1993；Pang,1989
Ostracode	Middle Spathian- Late Spathian	*Darwinula ingrata*	Middle Heshanggou Fm-Fugu, Shanxi	Pang, 1993；Pang,1989
Ostracode	Middle Spathian	*Darwinula recondita*	Lower Heshanggou Fm-Yima, Henan	Pang, 1993；Pang,1989

Ostracode	Middle Spathian- Late Spathian	*Darwinula accepta*	Middle Heshanggou Fm-Yima, Henan	Pang, 1993; Pang,1989
Ostracode	Middle Spathian- Late Spathian	*Darwinula promissa*	Middle Heshanggou Fm-Yima, Henan	Pang, 1993; Pang,1989
Ostracode	Middle Spathian- Late Spathian	*Darwinula zouzhigouensis*	Middle Heshanggou Fm-Yima, Henan	Pang, 1993; Pang,1989
Ostracode	Middle Spathian- Late Spathian	*Darwinula fragilis*	Upper Heshanggou Fm-Yaoxian, Shaanxi	Pang, 1993; Pang,1989
Ostracode	Middle Spathian- Late Spathian	*Darwinula oblonga*	Middle Heshanggou Fm-Hancheng, Shaanxi	Pang, 1993; Pang,1989
Ostracode	Middle Spathian- Late Spathian	*Darwinula cuspicata* var. *dilucida*	Middle Heshanggou Fm-Hancheng, Shaanxi	Pang, 1993; Pang,1989
Ostracode	Middle Spathian- Late Spathian	*Darwinula schneideri*	Middle Heshanggou Fm-Wupu, Shanxi	Pang, 1993; Pang,1989
Ostracode	Anisian	*Darwinula oblonga*	lower Middle Ermaying Fm-Yima, Henan	Pang and Whatley, 1990; Pang, 1989
Ostracode	Anisian	*Darwinula fragilis*	middle Upper Ermaying Fm-Tongchuan, Shaanxi	Pang and Whatley, 1990
Ostracode	Anisian	*Darwinula subovaliformis*	lower Middle Ermaying Fm-Yima, Henan;Upper Ermaying Fm-Dancheng, Henan	Pang and Whatley, 1990; Pang, 1989; Zhao, 1989
Ostracode	Anisian	*Darwinula accuminata*	middle Upper Ermaying Fm-Tongchuan, Shaanxi	Pang and Whatley, 1990
Ostracode	Anisian	*Darwinula alta*	Upper Ermaying Fm-Dancheng, Henan	Pang and Whatley, 1990
Ostracode	Anisian	*Lutkevichinella ornatula*	middle Upper Ermaying Fm-Tongchuan, Shaanxi	Pang and Whatley, 1990
Ostracode	Anisian	*Lutkevichinella brachicostata*	Lower-Upper Ermaying Fm	Pang and Whatley, 1990
Ostracode	Anisian	*Lutkevichinella minuta*	lower Middle Ermaying Fm-Yima, Henan ; middle Upper Ermaying Fm-Tongchuan, Shaanxi	Pang and Whatley, 1990; Pang, 1989
Ostracode	Anisian	*Lutkevichinella ansulca*	middle Upper Ermaying Fm-Tongchuan, Shaanxi	Pang and Whatley, 1990
Ostracode	Anisian	*Tungchuanina quadratiformis*	middle Upper Ermaying Fm-Tongchuan, Shaanxi	Pang and Whatley, 1990
Ostracode	Anisian	*Darwinula gerdae*	Lower- Upper Ermaying Fm-Tongchuan, Shaanxi	Pang, 1993
Ostracode	Anisian	*Darwinula tersiensis*	lower Middle Ermaying Fm-Yima, Henan	Pang, 1993;Pang, 1989
Ostracode	Anisian	*Darwinula subovaliformis*	lower Upper Ermaying Fm-Tongchuan, Shaanxi	Pang, 1993
Ostracode	Anisian	*Lutkevichinella ornata*	Lower-middle Upper Ermaying Fm-Tongchuan, Shaanxi	Pang, 1993

Ostracode	Anisian	*Lutkevichinella yimaensis*	lower Middle Ermaying Fm-Yima, Henan	Pang, 1993; Pang, 1989
Ostracode	Anisian	*Lutkevichinella sublongovata*	lower Middle Ermaying Fm-Yima, Henan	Pang, 1993; Pang, 1989
Ostracode	Anisian	*Lutkevichinella longovata*	Lower-middle Upper Ermaying Fm-Tongchuan, Shaanxi	Pang, 1993
Ostracode	Anisian	*Shansinella praecipua*	Upper Ermaying Fm-Tongchuan, Shaanxi	Pang, 1993
Ostracode	Anisian	*Shansinella gaoyadiensis*	Lower-middle Upper Ermaying Fm-Tongchuan, Shaanxi	Pang, 1993
Ostracode	Anisian	*Darwinula danchengensis*	Upper Ermaying Fm-Dancheng, Henan	Zhao, 1989
Ostracode	Anisian	*Darwinula comisa*	Upper Ermaying Fm-Dancheng, Henan	Zhao, 1989
Ostracode	Anisian	*Darwinula liulingchuanensis*	Upper Ermaying Fm-Dancheng, Henan; Upper Ermaying	Zhao, 1989; Pang, 1993
Ostracode	Anisian	*Darwinula postirecta*	Upper Ermaying Fm-Dancheng, Henan	Zhao, 1989
Ostracode	Anisian	*Darwinula* sp.2	Upper Ermaying Fm-Dancheng, Henan	Zhao, 1989
Ostracode	Anisian	*Darwinula schneideri*	middle Upper Ermaying Fm-Tongchuan, Shaanxi	Zhao, 1989

Table S4. **Conchostracan fossils data from Late Permian to Middle Triassic in North China.**

	Time	Species	Formation- Location	Reference
Conchostracan	Latest Changhsingian	*Falsisca eotriassica*	Upper Sunjiagou Fm-Tongchuan, Shaanxi	This study
Conchostracan	Latest Changhsingian	*Falsisca postera*	Upper Sunjiagou Fm-Tongchuan, Shaanxi	This study
Conchostracan	Latest Changhsingian	*Euestheria gutta*	Upper Sunjiagou Fm-Tongchuan, Shaanxi	This study
Conchostracan	Latest Changhsingian	*Euestheria oertlii*	Upper Sunjiagou Fm-Tongchuan, Shaanxi	This study
Conchostracan	Latest Changhsingian	*Huangheetheria longellipsa*	Upper Sunjiagou Fm-Tongchuan, Shaanxi	This study
Conchostracan	Latest Changhsingian	*Palaeolimnadia* sp.	Upper Sunjiagou Fm-Tongchuan, Shaanxi	This study
Conchostracan	Latest Changhsingian	*Hemicycloleaia sunanensis*	Upper Sunan Fm-Sunan, Gansu	Shan, 1984
Conchostracan	Latest Changhsingian	*Hemicycloleaia qinlongensis*	Upper Sunan Fm-Sunan, Gansu	Shan, 1984
Conchostracan	Latest Changhsingian	*Rostroleaia gansuensis*	Upper Sunan Fm-Sunan, Gansu	Shan, 1984
Conchostracan	Late Changhsingian	*Palaeolimnadia glabra*	Taohaiyingzi Fm-Shaowudameng, Inner Mongolia	Wang, 1984
Conchostracan	Late Changhsingian	*Palaeolimnadia rossica*	Taohaiyingzi Fm-Shaowudameng, Inner Mongolia	Wang, 1984
Conchostracan	Late Changhsingian	*Costestheria taohaiyingziensis*	Taohaiyingzi Fm-Shaowudameng, Inner Mongolia	Wang, 1984
Conchostracan	Late Changhsingian	*Costestheria scoliogabata*	Taohaiyingzi Fm-Shaowudameng, Inner Mongolia	Wang, 1984
Conchostracan	Late Changhsingian	*Pemphicyclus baiyintalaensis*	Taohaiyingzi Fm-Shaowudameng, Inner Mongolia	Wang, 1984
Conchostracan	Late Changhsingian	*Penphicyclus trochoides*	Taohaiyingzi Fm-Shaowudameng, Inner Mongolia	Wang, 1984
Conchostracan	Late Changhsingian	*Penphicyclus* cf. *arangastachus*	Taohaiyingzi Fm-Shaowudameng, Inner Mongolia	Wang, 1984
Conchostracan	Late Changhsingian	*Palaeolimnadiopsis deminuta*	Taohaiyingzi Fm-Shaowudameng, Inner Mongolia	Wang, 1984
Conchostracan	Late Changhsingian	*Rhyssestheria lampra*	Taohaiyingzi Fm-Shaowudameng, Inner Mongolia	Wang, 1984
Conchostracan	Late Changhsingian	*Rhyssestheria perfecta*	Taohaiyingzi Fm-Shaowudameng, Inner Mongolia	Wang, 1984
Conchostracan	Late Changhsingian	*Sphaerestheria* cf. *sibirica*	Taohaiyingzi Fm-Shaowudameng, Inner Mongolia	Wang, 1984
Conchostracan	Late Changhsingian	*Estherina aspred*	Taohaiyingzi Fm-Shaowudameng, Inner Mongolia	Wang, 1984
Conchostracan	Early Spathian	*Leptolimnadia shanxiensis*	Upper Liujiagou Fm-Jiaocheng, Shanxi	Liu, 1982

Conchostracan	Early Spathian	*Leptolimnadia jiaochengensis*	Upper Liujiagou Fm-Jiaocheng, Shanxi	Liu, 1982
Conchostracan	Early Spathian	*Palaeolimnadia komiana*	Upper Liujiagou Fm-Jiaocheng, Shanxi	Liu, 1982
Conchostracan	Early Spathian	*Palaeolimnadia chuanbeiensis*	Upper Liujiagou Fm-Jiaocheng, Shanxi	Liu, 1982
Conchostracan	Early Spathian	*Palaeolimnadia multilineata*	Upper Liujiagou Fm-Jiaocheng, Shanxi	Liu, 1982
Conchostracan	Early Spathian	*Palaeolimnadia contracta*	Upper Liujiagou Fm-Jiaocheng, Shanxi	Liu, 1982
Conchostracan	Early Spathian	*Lioestheria jiaochengensis*	Upper Liujiagou Fm-Jiaocheng, Shanxi	Liu, 1982
Conchostracan	Early Spathian	*Paleoleptestheria endybalica*	Upper Liujiagou Fm-Jiaocheng, Shanxi	Liu, 1982
Conchostracan	Early Spathian	*Loxomegaglypta jiaochengensis*	Upper Liujiagou Fm-Jiaocheng, Shanxi	Liu, 1982
Conchostracan	Late Spathian	*Glyptoasmussia quadrata*	Upper Heshanggou Fm-Hancheng, Shaanxi	Wang and Liu,1980
Conchostracan	Late Spathian	*Polygrapta subelliptica*	Upper Heshanggou Fm-Hancheng, Shaanxi	Wang and Liu,1980
Conchostracan	Late Spathian	*Polygrapta xuefengchuanensis*	Upper Heshanggou Fm-Hancheng, Shaanxi	Wang and Liu,1980
Conchostracan	Middle Spathian- Late Spathian	*Palaeolimnadia ovata*	Middle-Upper Heshanggou Fm-Hancheng, Shaanxi	Wang and Liu,1980
Conchostracan	Middle Spathian- Late Spathian	*Palaeolimnadia longovata*	Upper Heshanggou Fm-Hancheng, Shaanxi	Wang and Liu,1980
Conchostracan	Middle Spathian- Late Spathian	*Liolimnadia jiamijiawanensis*	Middle-Upper Heshanggou Fm-Hancheng, Shaanxi	Wang and Liu,1980
Conchostracan	Middle Spathian- Late Spathian	*Cornia guchengensis*	Middle-Upper Heshanggou Fm-Hancheng, Shaanxi	Wang and Liu,1980
Conchostracan	Middle Spathian- Late Spathian	*Gabonestheria clinotuberica*	Middle-Upper Heshanggou Fm-Hancheng, Shaanxi	Wang and Liu,1980
Conchostracan	Middle Spathian- Late Spathian	*Gabonestheria guchengchuanensis*	Middle-Upper Heshanggou Fm-Hancheng, Shaanxi	Wang and Liu,1980
Conchostracan	Late Spathian	*Aquilonoglypta clinoquadrata*	Upper Heshanggou Fm-Hancheng, Shaanxi	Wang and Liu,1980
Conchostracan	Late Spathian	*Aquilonoglypta xilougouensis*	Upper Heshanggou Fm-Hancheng, Shaanxi	Wang and Liu,1980
Conchostracan	Middle Spathian- Late Spathian	*Dictyostriaca subcyclata*	Middle-Upper Heshanggou Fm-Hancheng, Shaanxi	Wang and Liu,1980
Conchostracan	Late Spathian	*Loxomegaglypta sangerjiagouensis*	Upper Heshanggou Fm-Hancheng, Shaanxi	Wang and Liu,1980
Conchostracan	Middle Spathian- Late Spathian	*Leptonemia cyclata*	Middle-Upper Heshanggou Fm-Hancheng, Shaanxi	Wang and Liu,1980
Conchostracan	Middle Spathian- Late Spathian	*Diaplexa varidicta*	Middle-Upper Heshanggou Fm-Hancheng, Shaanxi	Wang and Liu,1980

Conchostracan	Late Spathian	*Lioestheria hanchengensis*	Upper Heshanggou Fm-Hancheng, Shaanxi	Wang and Liu,1980
Conchostracan	Late Spathian	*Palaeolimnadia magnapicalis*	Upper Heshanggou Fm-Hancheng, Shaanxi	Liu, 1995
Conchostracan	Late Spathian	*Eosolimnadia subquadrata*	Upper Heshanggou Fm-Hancheng, Shaanxi	Liu, 1995
Conchostracan	Late Spathian	*Eosolimnadia xingxianensis*	Upper Heshanggou Fm-Hancheng, Shaanxi	Liu, 1995
Conchostracan	Late Spathian	*Eosolimnadia shanxiensis*	Upper Heshanggou Fm-Hancheng, Shaanxi	Liu, 1995
Conchostracan	Late Spathian	*Triasestheria shanxiensis*	Upper Heshanggou Fm-Hancheng, Shaanxi	Liu, 1995
Conchostracan	Anisian	*Palaeolimnadia wupuensis*	Lower Ermaying Fm-Wupu, Shaanxi	Wu, 1991
Conchostracan	Anisian	*Palaeolimnadia megaformis*	Lower Ermaying Fm-Wupu, Shaanxi	Wu, 1991
Conchostracan	Anisian	*Palaeolimnadia concentrica*	Lower Ermaying Fm-Wupu, Shaanxi	Wu, 1991
Conchostracan	Anisian	*Palaeolimnadia glenoformis*	Lower Ermaying Fm-Wupu, Shaanxi	Wu, 1991
Conchostracan	Anisian	*Xiangxiella beifangensis*	Lower Ermaying Fm-Wupu, Shaanxi	Wu, 1991
Conchostracan	Anisian	*Protomonocarina reticalata*	Lower Ermaying Fm-Wupu, Shaanxi	Wu, 1991
Conchostracan	Anisian	*Protomonocarina zhangjiayanensis*	Lower Ermaying Fm-Wupu, Shaanxi	Wu, 1991
Conchostracan	Anisian	*Protomonocarina shanxiensis*	Lower Ermaying Fm-Wupu, Shaanxi	Wu, 1991
Conchostracan	Anisian	*Euestheria elliptica*	Lower Ermaying Fm-Wupu, Shaanxi	Wu, 1991
Conchostracan	Anisian	*Euestheria nitida*	Lower Ermaying Fm-Wupu, Shaanxi	Wu, 1991
Conchostracan	Anisian	*Euestheria brevielliptica*	Lower Ermaying Fm-Wupu, Shaanxi	Wu, 1991
Conchostracan	Anisian	*Brachystheria deflecta*	Lower Ermaying Fm-Wupu, Shaanxi	Wu, 1991
Conchostracan	Anisian	*Brachystheria ovata*	Lower Ermaying Fm-Wupu, Shaanxi	Wu, 1991
Conchostracan	Anisian	*Diaplexa qingshuiheensis*	Lower Ermaying Fm-Wupu, Shaanxi	Wu, 1991
Conchostracan	Anisian	*Diaplexa doliformis*	Lower Ermaying Fm-Wupu, Shaanxi	Wu, 1991
Conchostracan	Anisian	*Polygrapta wupuensis*	Lower Ermaying Fm-Wupu, Shaanxi	Wang and Liu,1980
Conchostracan	Anisian	*Lioestheria snbgibba*	Lower Ermaying Fm-Wupu, Shaanxi	Wang and Liu,1980

Conchostracan	Anisian	*Lioestheria zhangjiayanensis*	Lower Ermaying Fm-Wupu, Shaanxi	Wang and Liu,1980
Conchostracan	Anisian	*Lioestheria shensiensis*	Middle Ermaying Fm-Hancheng, Shaanxi	Wang and Liu,1980
Conchostracan	Anisian	*Brachystheria subdisca*	Middle Ermaying Fm-Hancheng, Shaanxi	Wang and Liu,1980
Conchostracan	Anisian	*Protomonocarina binoda*	Lower Ermaying Fm-Yuzhuang, Shanxi	Liu, 1984
Conchostracan	Anisian	*Trisitum mulsilineasum*	Lower Ermaying Fm-Yuzhuang, Shanxi	Liu, 1984
Conchostracan	Anisian	*Gabonestheria shanxiensis*	Lower Ermaying Fm-Yuzhuang, Shanxi	Liu, 1984
Conchostracan	Anisian	*Gabonestheria arcuata*	Lower Ermaying Fm-Yuzhuang, Shanxi	Liu, 1984
Conchostracan	Anisian	*Gabonestheria fusiformis*	Lower Ermaying Fm-Yuzhuang, Shanxi	Liu, 1984
Conchostracan	Anisian	*Palaeolimnadia pusilla*	Lower Ermaying Fm-Yuzhuang, Shanxi	Liu, 1984
Conchostracan	Anisian	*Palaeolimnadia lubrica*	Lower Ermaying Fm-Yuzhuang, Shanxi	Liu, 1984
Conchostracan	Anisian	*Sphaerestheria minuta*	Lower Ermaying Fm-Yuzhuang, Shanxi	Liu, 1984
Conchostracan	Anisian	*Anyuanestheria shanxiensis*	Lower Ermaying Fm-Yuzhuang, Shanxi	Liu, 1984
Conchostracan	Anisian	*Punctestheria minuta*	Lower Ermaying Fm-Qinxian, Shanxi	Liu, 1984
Conchostracan	Anisian	*Punctestheria cornuta*	Lower Ermaying Fm-Qinxian, Shanxi	Liu, 1984
Conchostracan	Anisian	*Punctestheria qinxianensis*	Lower Ermaying Fm-Qinxian, Shanxi	Liu, 1984
Conchostracan	Anisian	*Punctestheria* sp.	Lower Ermaying Fm-Qinxian, Shanxi	Liu, 1984

Table S5. **The volume size of *Darwinula* specimens from Late Changhsingian to Ansian.**

Number	Length	Highth	Thicknes	Volume	Time	Data source
1	760	410	375	7.786407114	Late Changhsingian-1	Dalongkou Section
2	618	380	365	7.651843334	Late Changhsingian-1	Dalongkou Section
3	704	384	350	7.694750326	Late Changhsingian-1	Dalongkou Section
4	733	400	360	7.742244864	Late Changhsingian-1	Dalongkou Section
5	750	300	275	7.51029361	Late Changhsingian-1	Dalongkou Section
6	630	390	355	7.659411907	Late Changhsingian-1	Dalongkou Section
7	713	278	220	7.358335404	Late Changhsingian-1	Dalongkou Section
8	600	280	245	7.333253764	Late Changhsingian-1	Dalongkou Section
9	742	400	355	7.741470647	Late Changhsingian-1	Dalongkou Section
10	723	410	375	7.764731819	Late Changhsingian-1	Dalongkou Section
11	765	400	355	7.754728177	Late Changhsingian-1	Dalongkou Section
12	630	310	265	7.432726515	Late Changhsingian-1	Dalongkou Section
13	600	300	310	7.465412597	Late Changhsingian-1	Dalongkou Section
14	601	300	260	7.389747472	Late Changhsingian-1	Dalongkou Section
15	661	380	355	7.668991807	Late Changhsingian-1	Dalongkou Section
16	630	305	270	7.433782551	Late Changhsingian-1	Dalongkou Section
17	545	298	255	7.335931344	Late Changhsingian-1	Dalongkou Section
18	690	310	300	7.526110437	Late Changhsingian-1	Dalongkou Section
19	791	400	331	7.738842866	Late Changhsingian-1	Dalongkou Section
20	672	385	355	7.681836753	Late Changhsingian-1	Dalongkou Section
21	700	365	350	7.670237347	Late Changhsingian-1	Dalongkou Section
22	765	350	313	7.642052215	Late Changhsingian-1	Dalongkou Section
23	720	320	300	7.558382127	Late Changhsingian-1	Dalongkou Section

24	695	385	365	7.708516796	Late Changhsingian-1	Dalongkou Section
25	660	350	300	7.559511632	Late Changhsingian-1	Dalongkou Section
26	705	325	305	7.563150715	Late Changhsingian-1	Dalongkou Section
27	666	380	355	7.672264577	Late Changhsingian-1	Dalongkou Section
28	730	343	313	7.612939715	Late Changhsingian-1	Dalongkou Section
29	585	298	250	7.358090537	Late Changhsingian-1	Dalongkou Section
30	655	327	285	7.50441231	Late Changhsingian-1	Dalongkou Section
31	667	267	230	7.331143329	Late Changhsingian-1	Dalongkou Section
32	407	200	170	6.859851724	Late Changhsingian-1	Dalongkou Section
33	660	270	220	7.312108778	Late Changhsingian-1	Dalongkou Section
34	555	210	175	7.028328724	Late Changhsingian-1	Dalongkou Section
35	771	310	270	7.528558234	Late Changhsingian-1	Dalongkou Section
36	810	352	310	7.665167774	Late Changhsingian-1	Dalongkou Section
37	600	285	255	7.358314689	Late Changhsingian-1	Dalongkou Section
38	630	310	280	7.456638672	Late Changhsingian-1	Dalongkou Section
39	690	300	275	7.474081437	Late Changhsingian-1	Dalongkou Section
40	780	330	295	7.599208956	Late Changhsingian-1	Dalongkou Section
41	630	300	265	7.418486076	Late Changhsingian-1	Dalongkou Section
42	600	310	280	7.435449373	Late Changhsingian-1	Dalongkou Section
43	700	350	320	7.61309446	Late Changhsingian-1	Dalongkou Section
44	630	300	275	7.434572896	Late Changhsingian-1	Dalongkou Section
45	600	250	225	7.247052175	Late Changhsingian-1	Dalongkou Section
46	700	300	275	7.480330386	Late Changhsingian-1	Dalongkou Section
47	700	300	275	7.480330386	Late Changhsingian-1	Dalongkou Section
48	800	400	375	7.797959644	Late Changhsingian-1	Dalongkou Section

49	600	250	225	7.247052175	Late Changhsingian-1	Dalongkou Section
50	620	380	350	7.635021728	Late Changhsingian-1	Dalongkou Section
51	650	330	305	7.534505534	Late Changhsingian-1	Dalongkou Section
52	600	280	250	7.342027688	Late Changhsingian-1	Dalongkou Section
53	750	400	385	7.781360382	Late Changhsingian-1	Dalongkou Section
54	650	250	225	7.281814281	Late Changhsingian-1	Dalongkou Section
55	825	400	375	7.811323605	Late Changhsingian-1	Dalongkou Section
56	600	300	275	7.413383597	Late Changhsingian-1	Dalongkou Section
57	700	350	320	7.61309446	Late Changhsingian-1	Dalongkou Section
58	550	275	250	7.29641379	Late Changhsingian-1	Dalongkou Section
59	775	375	350	7.726179412	Late Changhsingian-1	Dalongkou Section
60	700	400	380	7.745720026	Late Changhsingian-1	Dalongkou Section
61	650	350	325	7.58764316	Late Changhsingian-1	Dalongkou Section
62	625	300	275	7.431112364	Late Changhsingian-1	Dalongkou Section
63	650	300	275	7.448145703	Late Changhsingian-1	Dalongkou Section
64	725	325	300	7.56812102	Late Changhsingian-1	Dalongkou Section
65	800	300	275	7.538322333	Late Changhsingian-1	Dalongkou Section
66	550	250	225	7.209263614	Late Changhsingian-1	Dalongkou Section
67	600	250	230	7.256597493	Late Changhsingian-1	Dalongkou Section
68	550	250	225	7.209263614	Late Changhsingian-1	Dalongkou Section
69	647	290	253	7.395201197	Late Changhsingian-1	Dalongkou Section
70	700	360	345	7.657998034	Late Changhsingian-1	Dalongkou Section
71	605	305	285	7.439678472	Late Changhsingian-1	Dalongkou Section
72	654	380	365	7.676432607	Late Changhsingian-1	Dalongkou Section
73	770	410	385	7.803513709	Late Changhsingian-1	Dalongkou Section

74	690	390	365	7.71098496	Late Changhsingian-1	Dalongkou Section
75	685	325	295	7.536174346	Late Changhsingian-1	Dalongkou Section
76	654	325	300	7.523360762	Late Changhsingian-1	Dalongkou Section
77	720	360	335	7.657458202	Late Changhsingian-1	Dalongkou Section
78	577	311	290	7.435112598	Late Changhsingian-1	Dalongkou Section
79	685	371	350	7.667910923	Late Changhsingian-1	Dalongkou Section
80	630	381	355	7.649272276	Late Changhsingian-1	Dalongkou Section
81	670	350	320	7.594071223	Late Changhsingian-1	Dalongkou Section
82	630	360	335	7.599466255	Late Changhsingian-1	Dalongkou Section
83	650	450	435	7.823393525	Late Changhsingian-1	Dalongkou Section
84	900	300	275	7.589474856	Late Changhsingian-1	Dalongkou Section
85	500	275	250	7.255021105	Late Changhsingian-1	Dalongkou Section
86	600	257	214	7.238097332	Late Changhsingian-1	Dalongkou Section
87	655	245	205	7.235479121	Late Changhsingian-1	Dalongkou Section
88	683	329	280	7.518616468	Late Changhsingian-1	Dalongkou Section
89	672	264	216	7.302205349	Late Changhsingian-1	Dalongkou Section
90	625	287.5	237.5	7.348959878	Late Changhsingian-1	Dalongkou Section
91	575	300	262.5	7.374696805	Late Changhsingian-1	Dalongkou Section
92	600	275	200	7.237292338	Late Changhsingian-1	Dalongkou Section
93	425	212.5	175	6.917564311	Late Changhsingian-1	Dalongkou Section
94	343	171	157	6.684269451	Late Changhsingian-1	Dalongkou Section
95	275	175	163	6.612002506	Late Changhsingian-1	Dalongkou Section
96	525	200	188	7.012968969	Late Changhsingian-1	Dalongkou Section
97	500	250	220	7.158111092	Late Changhsingian-1	Dalongkou Section
98	417	200	175	6.8826352	Late Changhsingian-1	Dalongkou Section

99	390	181	162	6.77719688	Late Changhsingian-1	Dalongkou Section
100	660	330	300	7.533957528	Late Changhsingian-1	Dalongkou Section
101	650	300	300	7.485934264	Late Changhsingian-1	Dalongkou Section
102	600	300	270	7.405414667	Late Changhsingian-1	Dalongkou Section
103	500	275	235	7.228148958	Late Changhsingian-1	Dalongkou Section
104	650	300	265	7.432058883	Late Changhsingian-1	Dalongkou Section
105	600	250	225	7.247052175	Late Changhsingian-1	Dalongkou Section
106	600	300	300	7.451172158	Late Changhsingian-1	Dalongkou Section
107	800	250	250	7.417748402	Late Changhsingian-1	Dalongkou Section
108	650	350	300	7.552881053	Late Changhsingian-1	Dalongkou Section
109	500	300	250	7.292809665	Late Changhsingian-1	Dalongkou Section
110	500	250	250	7.213628419	Late Changhsingian-1	Dalongkou Section
111	500	200	200	7.019808393	Late Changhsingian- 2	Dalongkou Section
112	700	300	270	7.472361457	Late Changhsingian- 2	Dalongkou Section
113	450	300	260	7.264085514	Late Changhsingian- 2	Dalongkou Section
114	600	300	250	7.371990911	Late Changhsingian- 2	Dalongkou Section
115	700	250	250	7.359756455	Late Changhsingian- 2	Dalongkou Section
116	700	240	240	7.324298921	Late Changhsingian- 2	Dalongkou Section
117	650	250	230	7.291359599	Late Changhsingian- 2	Dalongkou Section
118	550	250	230	7.218808932	Late Changhsingian- 2	Dalongkou Section
119	600	350	300	7.518118947	Late Changhsingian- 2	Dalongkou Section
120	750	400	375	7.76993092	Late Changhsingian- 2	Dalongkou Section
121	700	300	275	7.480330386	Late Changhsingian- 2	Dalongkou Section
122	650	400	375	7.707783013	Late Changhsingian- 2	Dalongkou Section
123	650	280	265	7.40209566	Late Changhsingian- 2	Dalongkou Section

124	450	225	200	7.025203425	Late Changhsingian- 2	Dalongkou Section
125	850	450	420	7.924659128	Late Changhsingian- 2	Dalongkou Section
126	750	350	325	7.649791066	Late Changhsingian- 2	Dalongkou Section
127	650	300	275	7.448145703	Late Changhsingian- 2	Dalongkou Section
128	700	350	325	7.619827843	Late Changhsingian- 2	Dalongkou Section
129	550	280	265	7.329544992	Late Changhsingian- 2	Dalongkou Section
130	600	300	275	7.413383597	Late Changhsingian- 2	Dalongkou Section
131	650	280	265	7.40209566	Late Changhsingian- 2	Dalongkou Section
132	700	290	265	7.44952031	Late Changhsingian- 2	Dalongkou Section
133	600	300	275	7.413383597	Late Changhsingian- 2	Dalongkou Section
134	720	350	325	7.632062299	Late Changhsingian- 2	Dalongkou Section
135	600	250	225	7.247052175	Late Changhsingian- 2	Dalongkou Section
136	650	300	275	7.448145703	Late Changhsingian- 2	Dalongkou Section
137	600	250	225	7.247052175	Late Changhsingian- 2	Dalongkou Section
138	650	300	275	7.448145703	Late Changhsingian- 2	Dalongkou Section
139	840	360	330	7.717874124	Late Changhsingian- 2	Dalongkou Section
140	600	300	270	7.405414667	Late Changhsingian- 2	Dalongkou Section
141	443	261	235	7.152740072	Late Changhsingian- 2	Dalongkou Section
142	625	225	200	7.167870929	Late Changhsingian- 2	Dalongkou Section
143	570	270	240	7.286228259	Late Changhsingian- 2	Dalongkou Section
144	720	270	150	7.183565917	Late Changhsingian- 2	Dalongkou Section
145	720	375	330	7.668656102	Late Changhsingian- 2	Dalongkou Section
146	660	285	240	7.373378435	Late Changhsingian- 2	Dalongkou Section
147	660	300	255	7.421983768	Late Changhsingian- 2	Dalongkou Section
148	660	315	270	7.467996651	Late Changhsingian- 2	Dalongkou Section

149	600	270	240	7.308504654	Late Changhsingian- 2	Dalongkou Section
150	320	160	136	6.561587267	Late Changhsingian- 2	Dalongkou Section
151	615	240	195	7.177899367	Late Changhsingian- 2	Dalongkou Section
152	630	270	240	7.329693953	Late Changhsingian- 2	Dalongkou Section
153	720	300	270	7.484595913	Late Changhsingian- 2	Dalongkou Section
154	660	270	240	7.349897339	Late Changhsingian- 2	Dalongkou Section
155	600	270	240	7.308504654	Late Changhsingian- 2	Dalongkou Section
156	630	210	195	7.130372853	Late Changhsingian- 2	Dalongkou Section
157	495	210	180	6.990875396	Late Changhsingian- 2	Dalongkou Section
158	667	300	267	7.445777126	Late Changhsingian- 2	Dalongkou Section
159	700	300	283	7.493295363	Late Changhsingian- 2	Dalongkou Section
160	650	300	300	7.485934264	Late Changhsingian- 2	Dalongkou Section
161	600	275	275	7.375595036	Late Changhsingian- 2	Dalongkou Section
162	650	350	350	7.619827843	Late Changhsingian- 2	Dalongkou Section
163	800	400	365	7.78622124	Late Changhsingian- 2	Dalongkou Section
164	750	250	225	7.343962188	Late Changhsingian- 2	Dalongkou Section
165	600	270	250	7.326233421	Late Changhsingian- 2	Dalongkou Section
166	700	300	285	7.495842552	Late Changhsingian- 2	Dalongkou Section
167	500	200	185	6.985950126	Late Changhsingian- 2	Dalongkou Section
168	700	280	265	7.434280343	Late Changhsingian- 2	Dalongkou Section
169	720	320	305	7.565560712	Late Changhsingian- 2	Dalongkou Section
170	600	300	285	7.428895763	Late Changhsingian- 2	Dalongkou Section
171	650	280	265	7.40209566	Late Changhsingian- 2	Dalongkou Section
172	800	400	375	7.797959644	Late Changhsingian- 2	Dalongkou Section
173	600	450	425	7.778531092	Late Changhsingian- 2	Dalongkou Section

174	700	300	275	7.480330386	Late Changhsingian- 2	Dalongkou Section
175	660	270	255	7.376226278	Late Changhsingian- 2	Dalongkou Section
176	620	350	325	7.567121493	Late Changhsingian- 2	Dalongkou Section
177	650	320	300	7.513962987	Late Changhsingian- 2	Dalongkou Section
178	700	380	365	7.705952899	Late Changhsingian- 2	Dalongkou Section
179	650	300	280	7.45597104	Late Changhsingian- 2	Dalongkou Section
180	550	300	275	7.375595036	Late Changhsingian- 2	Dalongkou Section
181	650	450	425	7.813293198	Late Changhsingian- 2	Dalongkou Section
182	500	200	185	6.985950126	Late Changhsingian- 2	Dalongkou Section
183	580	280	265	7.352610297	Late Changhsingian- 2	Dalongkou Section
184	600	300	275	7.413383597	Late Changhsingian- 2	Dalongkou Section
185	600	320	305	7.486379466	Late Changhsingian- 2	Dalongkou Section
186	700	300	275	7.480330386	Late Changhsingian- 2	Dalongkou Section
187	705	240	210	7.269398051	Late Changhsingian- 2	Dalongkou Section
188	504	228	192	7.06244501	Late Changhsingian- 2	Dalongkou Section
189	650	250	225	7.281814281	Late Changhsingian- 2	Dalongkou Section
190	714	343	314	7.604700378	Late Changhsingian- 2	Dalongkou Section
191	700	375	337.5	7.666181483	Late Changhsingian- 2	Dalongkou Section
192	660	360	315	7.592935388	Late Changhsingian- 2	Dalongkou Section
193	690	315	270	7.487301806	Late Changhsingian- 2	Dalongkou Section
194	505	253	221	7.169582571	Late Changhsingian- 2	Dalongkou Section
195	650	375	350	7.649791066	Late Changhsingian- 2	Dalongkou Section
196	675	250	225	7.298204697	Late Changhsingian- 2	Dalongkou Section
197	675	313	275	6.513636536	Late Changhsingian- 2	Dalongkou Section
198	625	300	275	7.431112364	Late Changhsingian- 2	Dalongkou Section

199	700	275	250	7.40114914	Late Changhsingian- 2	Dalongkou Section
200	690	300	270	7.466112507	Late Changhsingian- 2	Dalongkou Section
201	600	315	270	7.426603966	Late Changhsingian- 2	Dalongkou Section
202	540	300	270	7.359657176	Late Changhsingian- 2	Dalongkou Section
203	645	285	240	7.363394214	Late Changhsingian- 2	Dalongkou Section
204	570	255	225	6.614215927	Late Changhsingian- 2	Dalongkou Section
205	660	315	285	7.491477747	Late Changhsingian- 2	Dalongkou Section
206	630	240	210	7.220549484	Late Changhsingian- 2	Dalongkou Section
207	615	210	180	7.085145313	Late Changhsingian- 2	Dalongkou Section
208	660	240	210	7.24075287	Late Changhsingian- 2	Dalongkou Section
209	347	142	126	6.511766762	Late Changhsingian- 2	Dalongkou Section
210	411	152	126	6.614834353	Late Changhsingian- 2	Dalongkou Section
211	720	345	300	7.591051244	Late Changhsingian- 2	Dalongkou Section
212	615	240	210	7.21008405	Late Changhsingian- 2	Dalongkou Section
213	450	183	167	6.857158472	Late Changhsingian- 2	Dalongkou Section
214	450	200	160	6.87714089	Late Changhsingian- 2	Dalongkou Section
215	650	250	230	7.291359599	Late Changhsingian- 2	Dalongkou Section
216	300	183	158	6.658713442	Latest Changhsingian- Earliest Induan	Dalongkou Section
217	300	150	120	6.451172158	Latest Changhsingian- Earliest Induan	Dalongkou Section
218	555	255	210	7.191830856	Latest Changhsingian- Earliest Induan	Dalongkou Section
219	480	240	195	7.070265488	Latest Changhsingian- Earliest Induan	Dalongkou Section
220	400	167	142	6.693954814	Latest Changhsingian- Earliest Induan	Dalongkou Section
221	433	250	217	7.089332606	Latest Changhsingian- Earliest Induan	Dalongkou Section
222	690	300	240	7.414959985	Latest Changhsingian- Earliest Induan	Dalongkou Section
223	400	217	167	6.878479241	Latest Changhsingian- Earliest Induan	Dalongkou Section

224	545	182	145	6.877901571	Latest Changhsingian- Earliest Induan	Dalongkou Section
225	460	200	160	6.886686208	Latest Changhsingian- Earliest Induan	Dalongkou Section
226	420	160	120	6.625328917	Latest Changhsingian- Earliest Induan	Dalongkou Section
227	541	259	224	7.214455734	Latest Changhsingian- Earliest Induan	Dalongkou Section
228	460	180	140	6.78293677	Latest Changhsingian- Earliest Induan	Dalongkou Section
229	480	260	220	7.157415664	Latest Changhsingian- Earliest Induan	Dalongkou Section
230	480	200	160	6.905169613	Latest Changhsingian- Earliest Induan	Dalongkou Section
231	500	240	200	7.098989639	Latest Changhsingian- Earliest Induan	Dalongkou Section
232	360	180	140	6.676481439	Latest Changhsingian- Earliest Induan	Dalongkou Section
233	300	200	200	6.797959644	Latest Changhsingian- Earliest Induan	Dalongkou Section
234	300	180	180	6.706444663	Latest Changhsingian- Earliest Induan	Dalongkou Section
235	250	150	150	6.468900924	Latest Changhsingian- Earliest Induan	Dalongkou Section
236	500	220	205	7.071924944	Latest Changhsingian- Earliest Induan	Dalongkou Section
237	420	170	155	6.762808308	Latest Changhsingian- Earliest Induan	Dalongkou Section
238	500	150	120	6.673020907	Latest Changhsingian- Earliest Induan	Dalongkou Section
239	300	175	125	6.535847714	Latest Changhsingian- Earliest Induan	Dalongkou Section
240	520	220	180	7.032476927	Latest Changhsingian- Earliest Induan	Dalongkou Section
241	467	150	117	6.630823227	Latest Changhsingian- Earliest Induan	Dalongkou Section
242	600	236	171	7.103398758	Latest Changhsingian- Earliest Induan	Dalongkou Section
243	557	257	214	7.205912649	Latest Changhsingian- Earliest Induan	Dalongkou Section
244	483	200	167	6.92590389	Latest Changhsingian- Earliest Induan	Dalongkou Section
245	600	231	185	7.12637544	Latest Changhsingian- Earliest Induan	Dalongkou Section
246	643	214	171	7.091969297	Latest Changhsingian- Earliest Induan	Dalongkou Section
247	415	185	154	6.790583338	Latest Changhsingian- Earliest Induan	Dalongkou Section
248	669	208	162	7.070050652	Latest Changhsingian- Earliest Induan	Dalongkou Section

249	429	171	129	6.694029288	Latest Changhsingian- Earliest Induan	Dalongkou Section
250	357	186	129	6.649610148	Latest Changhsingian- Earliest Induan	Dalongkou Section
251	627	218	164	7.06893318	Latest Changhsingian- Earliest Induan	Dalongkou Section
252	398	150	90	6.448995238	Latest Changhsingian- Earliest Induan	Dalongkou Section
253	596	250	198	7.188629856	Latest Changhsingian- Earliest Induan	Dalongkou Section
254	520	200	165	6.953295681	Latest Changhsingian- Earliest Induan	Dalongkou Section
255	600	220	185	7.106524057	Latest Changhsingian- Earliest Induan	Dalongkou Section
256	350	200	165	6.781360382	Latest Changhsingian- Earliest Induan	Dalongkou Section
257	400	200	165	6.839352329	Latest Changhsingian- Earliest Induan	Dalongkou Section
258	500	250	210	7.137907705	Latest Changhsingian- Earliest Induan	Dalongkou Section
259	650	270	235	7.334123381	Latest Changhsingian- Earliest Induan	Dalongkou Section
260	450	240	205	7.063956014	Latest Changhsingian- Earliest Induan	Dalongkou Section
261	600	200	165	7.015443588	Latest Changhsingian- Earliest Induan	Dalongkou Section
262	700	350	305	7.592244321	Latest Changhsingian- Earliest Induan	Dalongkou Section
263	500	100	85	6.347167328	Latest Changhsingian- Earliest Induan	Dalongkou Section
264	350	120	100	6.342027688	Latest Changhsingian- Earliest Induan	Dalongkou Section
265	400	150	120	6.576110894	Latest Changhsingian- Earliest Induan	Dalongkou Section
266	400	200	165	6.839352329	Latest Changhsingian- Earliest Induan	Dalongkou Section
267	420	170	135	6.702810378	Latest Changhsingian- Earliest Induan	Dalongkou Section
268	600	150	135	6.803354676	Latest Changhsingian- Earliest Induan	Dalongkou Section
269	300	150	120	6.451172158	Latest Changhsingian- Earliest Induan	Dalongkou Section
270	550	250	230	7.218808932	Latest Changhsingian- Earliest Induan	Dalongkou Section
271	600	200	175	7.040997692	Latest Changhsingian- Earliest Induan	Dalongkou Section
272	420	180	165	6.814784137	Latest Changhsingian- Earliest Induan	Dalongkou Section
273	500	200	175	6.961816446	Latest Changhsingian- Earliest Induan	Dalongkou Section

274	600	250	225	7.247052175	Latest Changhsingian- Earliest Induan	Dalongkou Section
275	603	275	220	7.280851084	Latest Changhsingian- Earliest Induan	Dalongkou Section
276	498	200	140	6.863165772	Latest Changhsingian- Earliest Induan	Dalongkou Section
277	605	260	212	7.241842981	Latest Changhsingian- Earliest Induan	Dalongkou Section
278	740	255	215	7.326988758	Latest Changhsingian- Earliest Induan	Dalongkou Section
279	738	250	200	7.285804764	Latest Changhsingian- Earliest Induan	Dalongkou Section
280	600	150	98	6.664246983	Latest Changhsingian- Earliest Induan	Dalongkou Section
281	255	100	55	5.865681268	Latest Changhsingian- Earliest Induan	Dalongkou Section
282	500	250	198	7.112353601	Latest Changhsingian- Earliest Induan	Dalongkou Section
283	550	250	190	7.135834697	Latest Changhsingian- Earliest Induan	Dalongkou Section
284	500	200	148	6.889040113	Latest Changhsingian- Earliest Induan	this study
285	600	288	240	7.336533378	Spathian	Su et al., 1980
286	576	288	240	7.318804611	Spathian	Su et al., 1980
287	864	408	336	7.792291581	Spathian	Su et al., 1980
288	912	433	360	7.871563633	Spathian	Su et al., 1980
289	720	360	240	7.512624637	Spathian	Su et al., 1980
290	648	312	192	7.307809226	Spathian	Su et al., 1980
291	528	264	192	7.146317476	Spathian	Su et al., 1980
292	600	240	216	7.211594641	Spathian	Su et al., 1980
293	672	264	264	7.389355524	Spathian	Su et al., 1980
294	672	264	264	7.389355524	Spathian	Su et al., 1980
295	624	288	216	7.307809226	Spathian	Su et al., 1980
296	648	264	216	7.286411082	Spathian	Su et al., 1980
297	312	168	144	6.596604766	Spathian	Su et al., 1980
298	408	120	96	6.39089104	Spathian	Su et al., 1980

299	650	290	240	7.374300994	Spathian	Pang, 1989
300	620	280	190	7.237081719	Spathian	Pang, 1989
301	620	260	190	7.204897036	Spathian	Pang, 1989
302	640	300	240	7.382290868	Spathian	Pang, 1989
303	790	420	320	7.744804758	Spathian	Pang, 1989
304	780	380	310	7.682018291	Spathian	Pang, 1989
305	670	300	240	7.402185697	Spathian	Pang, 1989
306	540	240	190	7.110137	Spathian	Pang, 1989
307	740	350	370	7.700279886	Spathian	Pang, 1989
308	750	380	310	7.664984952	Spathian	Pang, 1989
309	770	400	420	7.830578405	Spathian	Pang, 1989
310	790	410	420	7.852438636	Spathian	Pang, 1989
311	780	350	370	7.723142769	Spathian	Pang, 1989
312	850	460	460	7.973712987	Spathian	Pang, 1989
313	650	380	250	7.50941536	Spathian	Pang, 1989
314	500	350	240	7.342027688	Spathian	Pang, 1989
315	500	260	250	7.230661759	Spathian	Pang, 1989
316	420	220	180	6.939722874	Spathian	Pang, 1989
317	860	430	250	7.684685313	Spathian	Pang, 1989
318	598	324	218	7.210420191	Anisian	Zhao, 1989
319	598	312	218	7.228518413	Anisian	Zhao, 1989
320	572	338	208	7.251214262	Anisian	Zhao, 1989
321	598	312	234	7.25350237	Anisian	Zhao, 1989
322	611	338	234	7.266897449	Anisian	Zhao, 1989
323	598	312	208	7.307050105	Anisian	Zhao, 1989

324	650	364	260	7.507766486	Anisian	Zhao, 1989
325	624	338	234	7.412095545	Anisian	Zhao, 1989
326	611	364	208	7.323544282	Anisian	Zhao, 1989
327	624	364	260	7.490037719	Anisian	Zhao, 1989
328	650	364	235	7.463861	Anisian	Zhao, 1989
329	740	370	240	7.536423083	Anisian	Pang, 1989
330	660	350	230	7.444118214	Anisian	Pang, 1989
331	710	320	250	7.386231351	Anisian	Pang, 1989
332	860	480	420	7.396531307	Anisian	Pang, 1989
333	710	310	240	7.396531307	Anisian	Pang, 1989
334	650	310	170	7.25350237	Anisian	Pang, 1989
335	470	300	220	7.210420191	Anisian	Pang, 1989
336	500	350	230	7.441609682	Anisian	Pang, 1989
337	480	320	230	7.444118214	Anisian	Pang, 1989
338	470	290	250	7.251214262	Anisian	Pang, 1989
339	490	300	220	7.467741259	Anisian	Pang, 1989
340	500	310	250	7.473126733	Anisian	Pang, 1989
341	620	320	240	7.396531307	Anisian	Pang, 1989
342	600	310	250	7.386231351	Anisian	Pang, 1989
343	660	340	250	7.536423083	Anisian	Pang, 1989
344	640	310	240	7.957767377	Anisian	Pang, 1989

References

1. Banerjee, S., and Jeevankumar, S., 2005, Microbially originated wrinkle structures on sandstone and their stratigraphic context: Palaeoproterozoic Koldaha Shale, central India: Sedimentary Geology, v. 176, no. 1, p. 211-224.

2. Bosch, P., and Eriksson, P., 2008, A note on two occurrences of inferred microbial mat features preserved in the c. 2.1 Ga Magaliesberg Formation (Pretoria Group, Transvaal Supergroup) sandstones, near Pretoria, South Africa: South African Journal of Geology, v. 111, no. 2-3, p. 251-262.

3. Bouougri, E., and Porada, H., 2002, Mat-related sedimentary structures in Neoproterozoic peritidal passive margin deposits of the West African Craton (Anti-Atlas, Morocco): Sedimentary Geology, v. 153, no. 3, p. 85-106.

4. Bouougri, E. H., and Porada, H., 2007, Siliciclastic biolaminites indicative of widespread microbial mats in the Neoproterozoic Nama Group of Namibia: Journal of African Earth Sciences, v. 48, no. 1, p. 38-48.

5. Callow, R. H., Battison, L., and Brasier, M. D., 2011, Diverse microbially induced sedimentary structures from 1Ga lakes of the Diabaig Formation, Torridon Group, northwest Scotland: Sedimentary Geology, v. 239, no. 3, p. 117-128.

6. Chakraborty, P. P., Das, P., Saha, S., Das, K., Mishra, S. R., and Paul, P., 2012, Microbial mat related structures (MRS) from Mesoproterozoic Chhattisgarh and Khariar basins, Central India and their bearing on shallow marine sedimentation: Episodes, v. 35, no. 4, p. 513-523.

7. Cheng Z., W., Qu, L.F., Hou, J.P, and Li, P.X., 1983, Problems of Eumorphotes-bearing strata of the" Shihchienfeng Formation" in Qish-an, Shaanxi: Journal of Stratigraphy, v. 3, p. 000.

8. Cuadrado, D. G., Carmona, N. B., and Bournod, C., 2011, Biostabilization of sediments by microbial mats in a temperate siliciclastic tidal flat, Bahia Blanca estuary (Argentina): Sedimentary Geology, v. 237, no. 1, p. 95-101.

9. Erwin, D. H., 1994, The Permo-Triassic extinction: Nature, v. 367, p231-236.

10. Freytet, P., and Verrecchia, E. P., 1998, Freshwater organisms that build stromatolites: a synopsis of biocrystallization by prokaryotic and eukaryotic algae: Sedimentology, v. 45, no. 3, p. 535-563.

11. Gehling, J., 2000, Environmental interpretation and a sequence stratigraphic framework for the terminal Proterozoic Ediacara Member within the Rawnsley Quartzite, South Australia: Precambrian Research, v. 100, no. 1, p. 65-95.

12. Gehling, J. G., 1999, Microbial mats in terminal Proterozoic siliciclastics; Ediacaran death masks: Palaios, v. 14, no. 1, p. 40-57.

13. Gerdes, G., Claes, M., Dunajtschik-Piewak, K., Riege, H., Krumbein, W. E., and Reineck, H.-E., 1993, Contribution of microbial mats to sedimentary surface structures: Facies, v. 29, no. 1, p. 61-74.

14. He, B., 2009, Trace Fossils and Ichnofabrics in the Heshanggou Formation of Lacustrine Deposits, Jiyuan Area, Henan Province.

15. Heubeck, C., 2009, An early ecosystem of Archean tidal microbial mats (Moodies Group, South Africa, ca. 3.2 Ga): Geology, v. 37, no. 10, p. 931-934.

16. Kon'no, E., 1973, New species of Pleuromeia and Neocalamites from the Upper Scythian Bed in the Kitakami Massif, Japan: with a brief note on some Equisetacean Plants from the Upper Permian Bed in the Kitakami Massif.

17. Kryshtofovich, A., 1923, Pleuromeia and Hausmannia in Eastern Siberia, with a summary of recent contributions to the paleobotany of the region: American Journal of Science, no. 27, p. 200-208.

18. Lan, Z.W., and Chen, Z.Q., 2013, Proliferation of MISS-forming microbial mats after the late Neoproterozoic glaciations: Evidence from the Kimberley region, NW Australia: Precambrian Research, v. 224, p. 529-550.

19. Lin, L.Q., and Aimin, F., 1995, Shiqianfeng formation of Shanxi province: Journal of central China normal university (Natural sciences), p. 02.

20. Lin, J., Li, L., and Li, X.W., 2013, SHRIMP U-Pb zircon dating of the Triassic Ermaying and Tongchuan formations in Shanxi, China and its stratigraphic implications: Vertebrata palasiatica, v. 51, no. 002, p. 162-168.

21. Liu, S.W., 1995, Triassic continental strata and conchostracan faunas in China: Albertiana.

22. Liu, S.W., and He, Z.J., 2000, Marine conchostracans from the "Sunjiagou formation" of Qishan,

23. Mata, S. A., and Bottjer, D. J., 2009, The paleoenvironmental distribution of Phanerozoic wrinkle structures: Earth-Science Reviews, v. 96, no. 3, p. 181-195.

24. Meiyu, Z., 1989, Middle Triassic Ostracods from Ermaying Formation, Dancheng, Henan: Acta Micropalaeontologica Sinica, v. 2, p. 009.

25. Miller, M. F., and Smail, S. E., 1997, A semiquantitative field method for evaluating bioturbation on bedding planes: Palaios, v. 12, no. 4, p. 391-396.

26. Noffke, N., Gerdes, G., Klenke, T., and Krumbein, W., 1996, Microbially induced sedimentary structures-examples from modem sediments of siliciclastic tidal flats: Zbl Geol Palfiont Tell I, v. 1, p. 307-316.

27. Noffke, N., 2007, Microbially induced sedimentary structures in Archean sandstones: a new window into early life: Gondwana Research, v. 11, no. 3, p. 336-342.

28. Noffke, N., 2009, The criteria for the biogeneicity of microbially induced sedimentary structures (MISS) in Archean and younger, sandy deposits: Earth-Science Reviews, v. 96, no. 3, p. 173-180.

29. Noffke, N., Gerdes, G., and Klenke, T., 2003a, Benthic cyanobacteria and their influence on the sedimentary dynamics of peritidal depositional systems (siliciclastic, evaporitic salty, and evaporitic carbonatic): Earth-Science Reviews, v. 62, no. 1, p. 163-176.

30. Noffke, N., Hazen, R., and Nhleko, N., 2003b, Earth's earliest microbial mats in a siliciclastic marine environment (2.9 Ga Mozaan Group, South Africa): Geology, v. 31, no. 8, p. 673-676.

31. Noffke, N., Eriksson, K. A., Hazen, R. M., and Simpson, E. L., 2006b, A new window into Early Archean life: Microbial mats in Earth's oldest siliciclastic tidal deposits (3.2 Ga Moodies Group, South Africa): Geology, v. 34, no. 4, p. 253-256.

32. Noffke, N., and Paterson, D., 2008, Microbial interactions with physical sediment dynamics, and their significance for the interpretation of Earth's biological history: Geobiology, v. 6, no. 1, p. 1-4.

33. Noffke, N., Christian, D., Wacey, D., and Hazen, R. M., 2013, Microbially Induced Sedimentary Structures Recording an Ancient Ecosystem in the ca. 3.48 Billion-Year-Old Dresser Formation, Pilbara, Western Australia: Astrobiology, v. 13, no. 12, p. 1103-1124.

34. Pang, Q.Q, 1989, The Early-Middle Triassic Stratigraphy and Ostracoda From the Yima area in Henan Province: Journal of Shijiazhuang University of Economics, v. 3, p. 006.

35. Pang, Q.Q, and Whatley, R., 1990, The biostratigraphical sequence of Mesozoic non-marine ostracod assemblages in northern China, Ostracoda and Global Events, Springer, p. 239-250.

36. Pang, Q.Q, 1993, The nonmarine Triassic and ostracoda in northern China: New Mexico Museum of Natural History and Science Bulletin, v. 3, p. 383-392.

37. Porada, H., and Bouougri, E. H., 2007, Wrinkle structures—a critical review: Earth-Science Reviews, v. 81, no. 3, p. 199-215.

38. Porada, H., Ghergut, J., and Bouougri, E. H., 2008, Kinneyia-type wrinkle structures—critical review and model of formation: Palaios, v. 23, no. 2, p. 65-77.

39. Prave, A., 2002, Life on land in the Proterozoic: Evidence from the Torridonian rocks of northwest Scotland: Geology, v. 30, no. 9, p. 811-814.

40. Qu, L.F., 1982, The palynological assemblage from the Liujiagou formation of Jiaocheng, Shanxi: Bulletion of Chinese Academy of Geological Sciences, v. 4, p. 83-93.

41. Qi, Y., Hu, B., Zhang, G., and Gong, Y., 2007, Ichnofabrics and Their Environmental Interpretation from Middle Triassic Youfangzhuang Formation, Jiyuan Region, Western Henan Province: Acta Sedimentologica Sinica, v. 25, no. 3, p. 372.

42. Retallack, G. J., 1997, Earliest Triassic origin of Isoetes and quillwort evolutionary radiation: Journal of Paleontology, p. 500-521.

43. Sarkar, S., Bose, P. K., Samanta, P., Sengupta, P., and Eriksson, P. G., 2008, Microbial mat mediated structures in the Ediacaran Sonia Sandstone, Rajasthan, India, and their implications for Proterozoic sedimentation: Precambrian Research, v. 162, no. 1, p. 248-263.

44. Schieber, J., 1998, Possible indicators of microbial mat deposits in shales and sandstones: examples from the Mid-Proterozoic Belt Supergroup, Montana, USA: Sedimentary Geology, v. 120, no. 1, p. 105-124.

45. Seckbach, J., and Oren, A., 2010, Microbial mats: modern and ancient microorganisms in stratified systems, Springer.

46. Shen, Y.B., 1984, Occurrence of Permian leaid conchostracans in China and its palaeogeographical significance: Acta Palaeontologica Sinica, v. 4, p. 011.

47. Shikama, T., Kamei, T., and Murata, M., 1978, Early Triassic Ichthyosaurus, Utatsusaurus hataii gen. et sp. nov., from the Kitakami massif, Northeast Japan.

48. Ouyang, S., and Zhang, Z.L., 1982, Early Triassic palynological assemblage in Dengfeng, northwestern Henan: Acta Palaeontol. Sin, v. 21, no. 6, p. 685-696.

49. Ouyang, S., and Norris, G., 1988, Spores and pollen from the Lower Triassic Heshanggou Formation, Shaanxi Province, North China: Review of Palaeobotany and Palynology, v. 54, no. 3, p. 187-231.

50. Shuwen, L., 1982, On the Occurrence of Conchostracans in Liujiagou Formation in Shanxi Province: Acta Geol. Sin, v. 3, p. 264-269.

51. Simpson, E. L., Heness, E., Bumby, A., Eriksson, P. G., Eriksson, K. A., Hilbert-Wolf, H. L., Linnevelt, S., Malenda, H. F., Modungwa, T., and Okafor, O., 2013, Evidence for 2.0 Ga continental microbial mats in a paleodesert setting: Precambrian Research, v. 237, p. 36-50.

52. Thomas, K., Herminghaus, S., Porada, H., and Goehring, L., 2013, Formation of Kinneyia via shear-induced instabilities in microbial mats: Philosophical Transactions of the Royal Society A: Mathematical, Physical and Engineering Sciences, v. 371, no. 2004, p. 20120362.

53. Wu, T.Y., 1991, Conchostracan assemblage from bottom of Ermaying formation, Shaanxi: Acta Palaeontologica Sinica, v. 30, no. 5, p. 630-642.

54. Wang, W.L., 1984, Late Permian conchostracan from the Taohaiyingzi formation in Juudmeng, Innermongo [J]: Acta Palaeontologica Sinica, v. 1.

55. Wang, B., 1988, Division and correlation of upper Permian—lower Triassic in middle part of Henan and discussion on some geological topics: Expermental Petroleum Geology, v. 10, no. 2, p. 142-158.

56. Wang, Z.Q., and Wang, L.X., 1990, Late Early Triassic fossil plants from upper part of the Shiqianfeng Group in North China: Shanxi Geology, v. 5, no. 2, p. 97-154.

57. Wang, Z.Q., and Wang, L.X., 1986, Late Permian fossil plants from the lower part of the Shiqianfeng (Shihchienfeng) group in North China: Bull Tianjin Inst Geol Min Res, v. 15, p. 1-80.

58. Wang, Z.Q., 1996, Recovery of vegetation from the terminal Permian mass extinction in North China: Review of Palaeobotany and Palynology, v. 91, no. 1, p. 121-142.

59. Wang, Z.Q., and Wang, L.X., 1982, A new species of the lycopsid Pleuromeia from the Early Triassic of Shanxi, China, and its ecology: Palaeontology, v. 25, no. 1, p. 215-225.

60. Wang, Z.Q., and Wang, L.X., 1990, New plant assemblages from the bottom of the Mid-Triassic Ermaying Formation: Shanxi Geology, v. 5, no. 4, p. 303-318.

61. Wang, R.N, 1982, Permian System of East Henan: Journal of China University of Mining & Technology, v. 2, p. 006.

62. Wang, R.N, 1997, New advance of the study of the Shiqianfeng formation in western China: Geological Review, v. 2, p. 015.

63. Xie, Z.M., Wang, L.X., and Wang, Z.Q., 1978, On the occurrence of Pleuromeia from the Qinshui basin in Shanxi province: Acta Palaeontologica Sinica, v. 2, p. 007.

64. Yang, W., Tian, J. C., Zhu, Y. T., and Wang, F., 2009, The storm deposits in the Upper Permian Shiqianfeng Formation in the Dengfeng region, Henan: Sedimentary Geology and Tethyan Geology, v. 1, p. 011.

65. Yin, H.F., Yang, Z.Y., and Lin, H.M., 1979, Marine Triassic faunas from Shiqianfeng group in the Weihe river basin, Shaanxi province: Acta Palaeontologica Sinica, v. 5, p. 004.

66. Zhu, R.K., Deng, S.H., Xu, H.X., and Guo, H.L., 2007, Research Institute of Petroleum Exploration and Development, PetroChina, B eijing 100083; Lithofacies palaeogeography of the Permian in northern China [J]: Journal of Palaeogeography, v. 9, no. 2, p. 133-142.

67. Zhang, H.Q., Liu, Y.H., and Lin, D.C., 1987, The discovery of a plant fossil assemblage in the Shiqianfeng formation in Yiyang, Henan, and its significence: Regional Geology of China, v. 4, p. 383-384.

68. Zhang K., 1991, Diachronism of the Sunjiagou Formation—A discussion: Regionl Geology of China, v. 3, p. 221-228.

69. Zhou, T.S., and Zhou, H.Q., 1983, Triassic nonmarine strata and flora of China: Bull. AOGS, v. 5, p. 95-110.
